# Controlling Intramolecular Förster Resonance Energy Transfer and Singlet Fission in a Subporphyrazine–Pentacene Conjugate by Solvent Polarity

**DOI:** 10.1002/anie.202011197

**Published:** 2020-11-18

**Authors:** David Guzmán, Ilias Papadopoulos, Giulia Lavarda, Parisa R. Rami, Rik R. Tykwinski, M. Salomé Rodríguez‐Morgade, Dirk M. Guldi, Tomás Torres

**Affiliations:** ^1^ Departamento de Química Orgánica Universidad Autónoma de Madrid Cantoblanco 28049 Madrid Spain; ^2^ IMDEA-Nanociencia Campus de Cantoblanco 28049 Madrid Spain; ^3^ Institute for Advanced Research in Chemical Sciences (IAdChem) Universidad Autónoma de Madrid Cantoblanco 28049 Madrid Spain; ^4^ Department of Chemistry and Pharmacy Interdisciplinary Center for Molecular Materials (ICMM) Friedrich-Alexander-Universität Erlangen-Nürnberg Egerlandstr. 3 91058 Erlangen Germany; ^5^ Department of Chemistry University of Alberta Edmonton Alberta T6G 2G2 Canada

**Keywords:** Förster resonance energy transfer, intramolecular singlet fission, subporphyrazine

## Abstract

Due its complementary absorptions in the range of 450 and 600 nm, an energy‐donating hexaaryl‐subporphyrazine has been linked to a pentacene dimer, which acts primarily as an energy acceptor and secondarily as a singlet fission enabler. In the corresponding conjugate, efficient intramolecular Förster resonance energy transfer (i‐FRET) is the modus operandi to transfer energy from the subporphyrazine to the pentacene dimer. Upon energy transfer, the pentacene dimer undergoes intramolecular singlet fission (i‐SF), that is, converting the singlet excited state, via an intermediate state, into a pair of correlated triplet excited states. Solvatochromic fluorescence of the subporphyrazine is a key feature of this system and features a red‐shift as large as 20 nm in polar media. Solvent is thus used to modulate spectral overlap between the fluorescence of subporphyrazine and absorption of the pentacene dimer, which controls the Förster rate constant, on one hand, and the triplet quantum yield, on the other hand. The optimum spectral overlap is realized in xylene, leading to Förster rate constant of 3.52×10^11^ s^−1^ and a triplet quantum yield of 171 % ±10 %. In short, the solvent polarity dependence, which is a unique feature of subporphyrazines, is decisive in terms of adjusting spectral overlap, ensuring a sizable Förster rate constant, and maximizing triplet quantum yields. Uniquely, this optimization can be achieved without a need for synthetic modification of the subporphyrazine donor.

## Graphical abstract

The solvatochromic fluorescence properties of a subporphyrazine are used to control the rate of intramolecular Förster resonance energy transfer (i‐FRET) in a subporphyrazine–pentacene conjugate (**SubPzPnc_2_**) that undergoes intramolecular singlet fission (i‐SF). A simple selection of solvent serves to optimize the triplet quantum yield by enhancing the spectral overlap between the fluorescence of the SubPz and the absorption of the Pnc dimer.

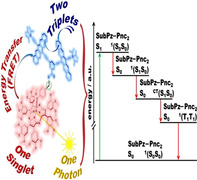

## Introduction

The process of transforming a singlet excited state (S_1_S_0_) into a correlated triplet excited state ^1^(T_1_T_1_), after the initial absorption of one photon, is known as singlet fission (SF) and possesses enormous potential for boosting the performance of single junction solar cells. By implementing SF in current solar cells, the Shockley‐Queisser limit, which is the theoretical limit of power conversion efficiency, could be increased from approximately 32 % to 45 %.[^1^, ^2^, ^3^] As such, major efforts have been conducted throughout the last decade with the trust to identify, characterize, and optimize materials for SF.[^1^, ^4^, ^5^, ^6^, ^7^, ^8^, ^9^, ^10^, ^11^]

In order for a given material to undergo SF, certain criteria must be met. To create a thermodynamic driving force, the energy of the first singlet excited state has to match, or be slightly larger than twice the energy of the first triplet excited state E(S_1_) ≥ 2E(T_1_). Furthermore, the energy level of the second triplet excited state should exceed twice the energy of the first triplet excited state, E(T_2_) >2E(T_1_) to avoid formation of higher triplet excited states from triplet‐triplet annihilation.[^1^, ^5^, ^12^] From an electronic coupling perspective, optimal communication between the individual chromophores involved in SF needs to be achieved. This is realized by controlling the distances and orbital overlap through crystal packing in the solid state, by high concentrations in solution, or by constructing covalently‐linked dimeric architectures.[^7^, ^10^, ^12^, ^13^, ^14^, ^15^, ^16^]

The mechanism of the singlet to triplet transformation is at the heart of SF and remains ardently investigated since there are numerous variables to be considered. A crucial factor that allows the formation of the single correlated triplet excited state ^1^(T_1_T_1_) is, in general, that the overall singlet multiplicity is maintained. As a result, SF is spin allowed, fast, and efficient. In stark contrast, the more prevalent intersystem crossing (ISC) process is spin forbidden, slow, and inefficient. Mechanistically, two main pathways have been identified for SF. On one hand is the direct, one‐step pathway, which proceeds without the formation of an intermediate state. On the other hand is the indirect, two‐step pathway, which includes the generation and population of an intermediate state. It facilitates the formation of ^1^(T_1_T_1_) by coupling to an energetically accessible charge transfer (CT) state.[^12^, ^17^, ^18^, ^19^, ^20^, ^21^, ^22^, ^23^, ^24^, ^25^, ^26^] This intermediate CT state may either be virtual, in form of a super‐exchange mechanism, or real, depending on its energy level with respect on the singlet and triplet excited states. Which of the two mechanisms prevails, depends on parameters such as electronic coupling, orientation of the chromophores, etc. A clear guideline to predict the mechanism is still debated and investigated to this date.

In terms of SF‐materials, acenes and, especially, pentacenes have evolved as the prototype. A plethora of pentacene‐based dimers that utilize a diverse library of linkers have been studied in recent years. At the forefront have been investigations regarding the effects of the linker on the (S_1_S_0_)‐to‐^1^(T_1_T_1_) transformation, its mechanism, the subsequent decoherence of ^1^(T_1_T_1_), triplet quantum yields (TQYs), and so on.[^16^, ^24^, ^27^, ^28^, ^29^, ^30^, ^31^, ^32^, ^33^, ^34^]

**Figure 1:**
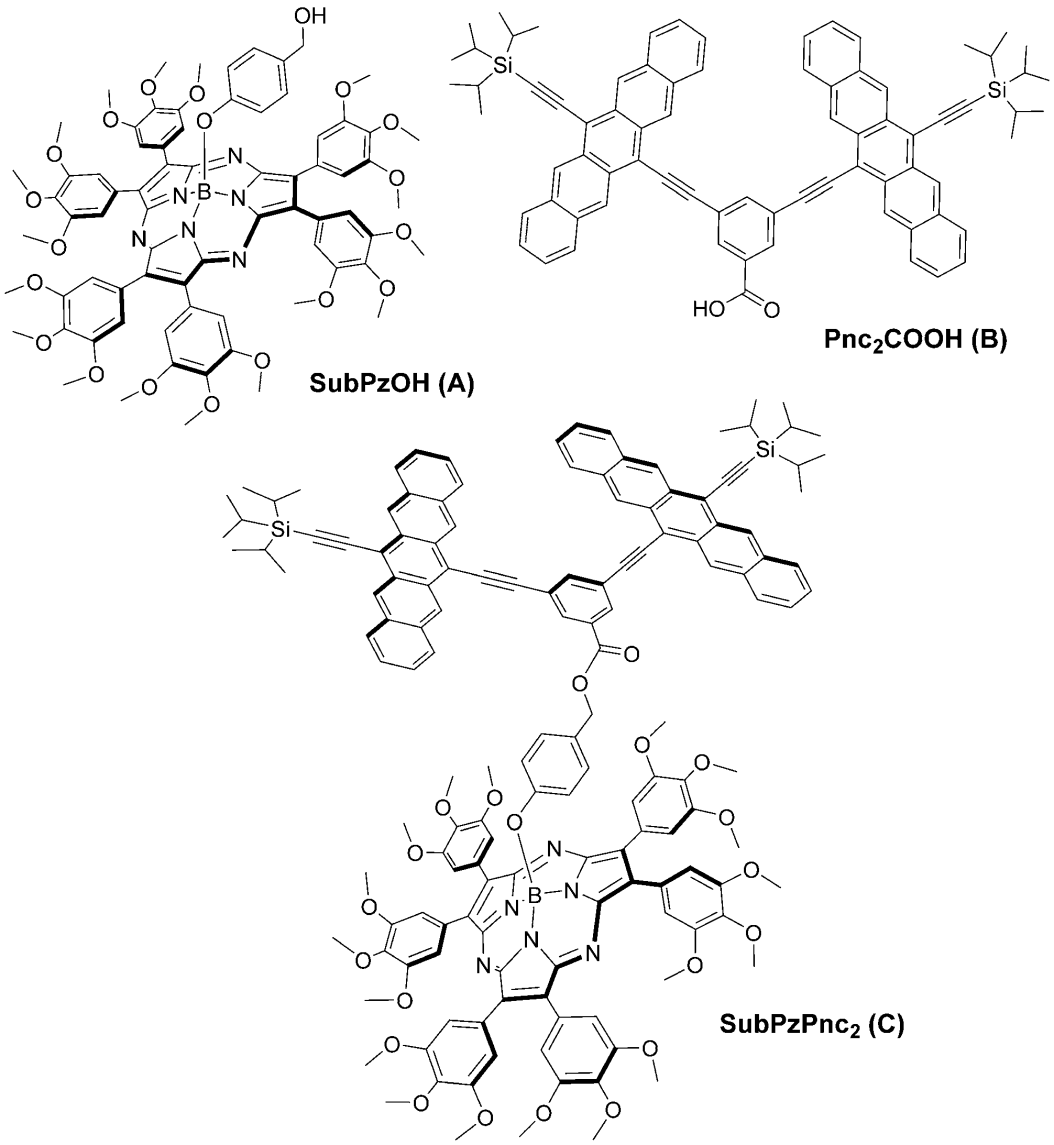
Chemical structures of **SubPzPnc_2_ (C)** and its precursors, namely **SubPzOH (A)** and **Pnc_2_COOH (B)**, used as reference compounds in this work.

Attention to date has remained focused on novel SF‐materials, their SF efficiencies, and how to manipulate their dynamics, as well as yields via tuning of coupling through linkers. Significant challenges remain with respect to improving materials that undergo SF in terms of, for example, versatility and panchromatic light harvesting. Notable efforts in this direction include the use of subphthalocyanine and porphyrazine conjugates, namely **SubPcPnc_2_** and **ZnPzPnc_2_**, respectively. SubPc and ZnPz served as energy antennae and donors that mediate their excited state energies via intramolecular Förster resonance energy transfer (i‐FRET) to Pnc_2_.[^35^, ^36^] By virtue of complementary absorptions, especially across the visible range, i‐FRET facilitates the overall efficiency of intramolecular SF (i‐SF). Notably, the choice of the respective energy donor dictates the spectral overlap (*J*) between fluorescence of the energy donor and absorption of the energy acceptor. Unfortunately, the use of neither SubPc nor ZnPz facilitates modification and/or fine‐tuning of *J* without committing to the time‐consuming process of synthetically altering the energy donor. As a result, the panchromatic absorptions of SubPc and ZnPz often remain unoptimized, especially within the 400 to 600 nm range. The work reported herein circumvents the need for synthetic alterations through the concept of fine‐tuning the emission of the antennae, and thus *J*, using solvent polarity. This has been done by opting for a conjugate comprising a subporphyrazine (SubPz) and Pnc_2_ yielding **C** (Figure 1).

SubPzs[^37^, ^38^] are a family of tripyrrolic, 14 π‐electron, aromatic porphyrinoids of conical shape. SubPzs are far more than just SubPc derivatives lacking the fused benzene rings. They are unique chromophores and show outstanding tunability of their properties by, for example, peripheral functionalization. The versatility of SubPzs in terms of their electronic structure stems from the combination of three structural features, namely electron‐deficient boron(III) central ions, meso‐aza bridges, and non‐fused pyrrolic units.^[39]^ The latter point is of great importance, as it allows the direct attachment of peripheral substituents to the β‐positions of those pyrroles that constitute the macrocycle. In this way, the substituents exert much stronger influence on the aromatic heteroannulene than in SubPcs, where the substituents are appended through the fused benzene rings.[^38^, ^39^, ^40^] As a matter of fact, axial substitution of SubPzs by nucleophiles was thought to reflect stronger interactions of the peripheral substituents with the macrocycle than in the case of SubPcs.[^38^, ^41^] Difficulties associated with the synthesis of SubPzs can be, however, a major drawback. Nevertheless, our recently established peripheral arylation and vinylation methods have allowed preparation of a library of SubPzs, and based on these previous studies,[^39^, ^40^] we envisioned that a SubPz with six peripheral 3,4,5‐trimethoxyphenyl substituents, such as **A**, should complement Pnc_2_. In doing so, this SubPz adds strong absorptions between 450 and 550 nm, a range where the Pnc_2_ shows little or no absorption, as well as a broad fluorescence at a 668 nm maximum, which matches quite well the strongest absorption of the Pnc_2_.^[39]^ Other type of peripheral substitution, such as sulfanyl groups, provides suitable absorption properties, but shifts the SubPz fluorescence band to lower energies (λ_em_=682 nm).^[39]^ Therefore, we report here on the synthesis of **C** (Figure 1), in which SubPz and Pnc_2_ are connected through the axial position of SubPz. Axial substitution induces little, if any, changes to the optical spectra of SubPzs.[^41^, ^42^] In other words, linking the Pnc_2_ preserves the absorption/fluorescence of the SubPz and, in turn, maintains its electronic complementarity to the Pnc_2_. Photophysical assays corroborate that SubPz‐centered i‐FRET to Pnc_2_ followed by a Pnc_2_‐centered i‐SF is operative and can be moderated through solvent choice.

## Results and Discussion

### Synthesis

**Scheme 1:**
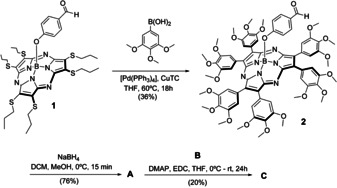
Preparation of **SubPzPnc_2_ (C)**.

**C** was synthesized by esterification of a bispentacene containing a carboxylic acid function (**B**) and a suitable SubPz derivative, **A**, endowed with an alcohol moiety. Formyl‐SubPz **2** (Scheme 1) is the key intermediate for the synthesis of **A** and was prepared in 36 % yield by the general hexaarylation procedure,^[40]^ starting from the formyl‐substituted, hexasulfanyl‐SubPz **1**. The reduction of **2** was performed with NaBH_4_ and afforded **A** in 76 % yield. Finally, treatment of **A** with **B** in THF, in the presence of dimethylaminopyridine (DMAP) and 1‐ethyl‐3‐(3‐dimethylaminopropyl)carbodiimide (EDC) provided **C** in 20 % yield.

### Photophysics

The optical spectra of known hexakis‐(3,4,5‐trimethoxyphenyl)SubPzs^[39]^ show three main strong absorptions, two of which appear at around 280 and 560 nm and are assigned to Soret and Q‐bands, respectively. A third feature at ca. 450 nm has been attributed to a CT‐like band arising from the strong interaction of the peripheral aryl groups with the macrocyclic core.[^39^, ^40^, ^43^]

**Figure 2:**
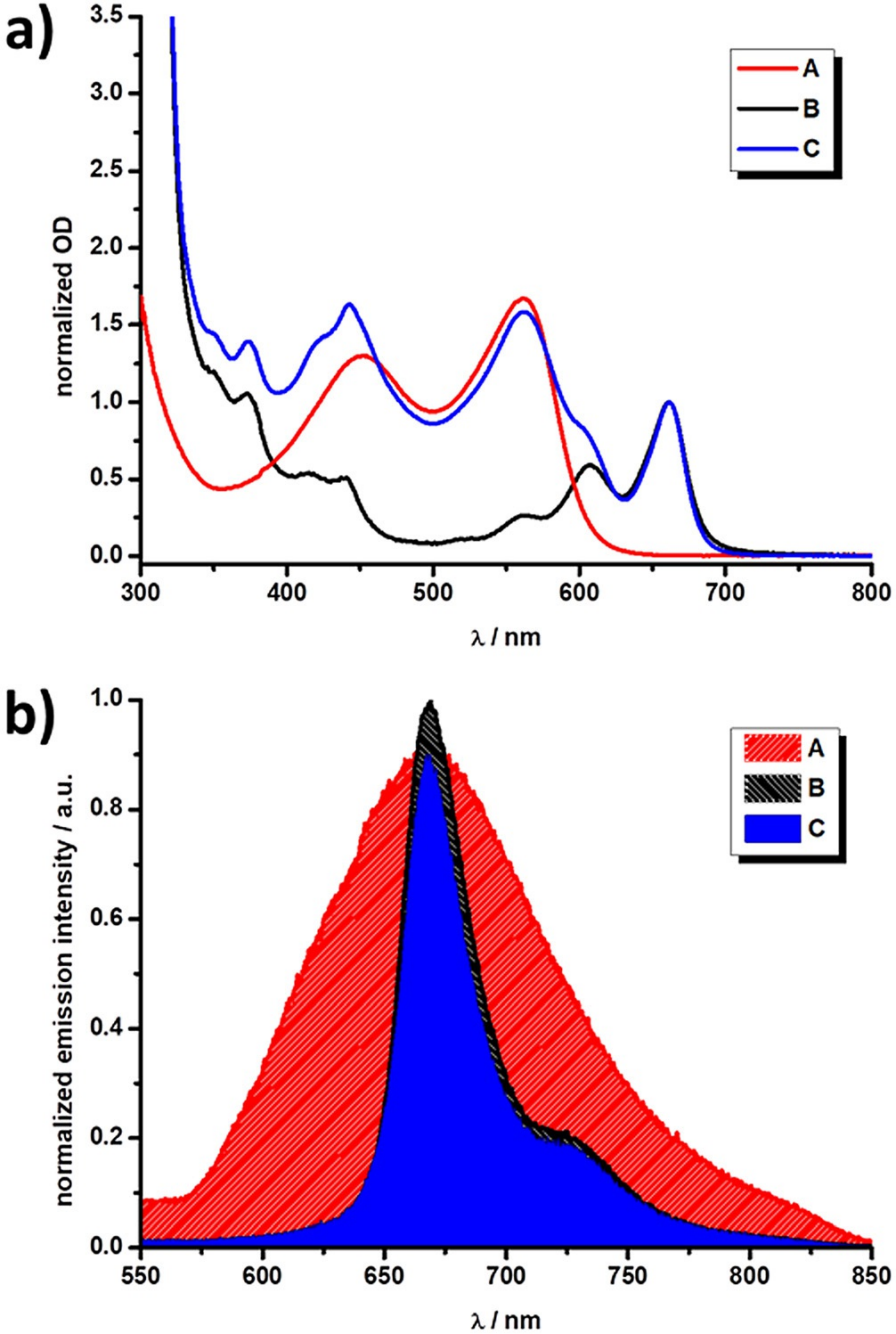
(a) Normalized absorption spectra of **A** (red), **B** (black), and **C** (blue) in xylene. (b) Normalized fluorescence spectra of **A** (red), **B** (black), and **C** (blue) in xylene. Normalization in (a): The **B** centered absorption (≈660 nm) was used as a point of normalization for **B** and **C**, whereat the intensity of the **A** centered maximum (≈560 nm) was compared to **C**. Normalization in (b): The **B** centered fluorescence (≈670 nm) was used as a point of normalization, thus highlighting the comparatively lower *Φ*_F_ of **A** and **C**.

Steady‐state absorption measurements outline the ground state characteristics of **A** and **B**, individually, and their interactions within **C. B** features the well‐known fingerprint absorptions of pentacene and its derivatives. In particular, maxima evolve between 300 and 450 nm, as well as between 500 and 750 nm. As a complement, **A** exhibits broad CT and Q‐band features at roughly 450 and 560 nm, respectively, which are also present in **C**. As a matter of fact, the features of **C** are effectively the linear superposition of the individual parts of the conjugate, which confirms the absence of ground state interactions between SubPz and Pnc_2_ (Figure 2). Regardless of the solvent (xylene, toluene, anisole, and benzonitrile), no significant shifts were noted in the steady‐state absorptions. A comparison of the extinction coefficients in the respective solvents is given in the Supporting Information in Figures S23–S24. A comparison of panchromatic absorption presented by the conjugates **C** and **ZnPzPnc_2_**, from previous works[^35^, ^36^] conveys the advantage of SubPzs as energy donor. Namely, broad absorptions and high extinction coefficients in the 400 to 600 nm lead to an overall increase in absorption by roughly 85 % relative to **B**, which is superior to both SubPc (65 %) and ZnPz (51 %) as donors.

Steady‐state fluorescence measurements shed light on the excited state interactions between the donor SubPz and acceptor Pnc_2_ groups of **C**. Starting again with **B**, excitation as 610 nm results in fluorescence, which is a mirror image of the absorption, with maxima at approximately 670 and 725 nm. The fluorescence quantum yield (*Φ*_F_) is around 2 %, regardless of the solvent. Excitation of **A** at 540 nm leads to broad and featureless fluorescence, and the emission maximum shifts from ca. 665 to 690 nm as the solvent polarity is increased. Moreover, *Φ*_F_ for **A** is dependent on solvent polarity, with values that range from 2.4 % in xylene to <1.0 % in benzonitrile. Such low values of *Φ*_F_ for **A** might, however, create a bottleneck for FRET, as higher *Φ*_F_ of the energy donor, in general, benefit efficient energy transfer.

Focusing on **C** reveals a fluorescence pattern that closely resembles that of **B**, with *Φ*_F_ close to the 2 % of **B**. The lack of SubPz centered fluorescence in the conjugate derives from strong quenching, which is subsequently coincident with the residual Pnc_2_ centered fluorescence. This results from the fact that the fluorescence of **A** and **B** strongly overlap (Figure 2 and S22). Overall, the steady‐state fluorescence measurements document efficient interactions between the SubPz and the Pnc_2_ moieties in the excited state. This has been further documented in time‐correlated single photon counting (TCSPC) measurements. Upon excitation into the Q‐band of the SubPz and the detection of the Pnc_2_ centered fluorescence, intramolecular Förster resonance energy transfer (i‐FRET) is confirmed. In particular, excited state energy is efficiently transferred from the singlet excited state of the SubPz to the Pnc_2_ unit in **C**. In addition, the fluorescence lifetime of the SubPz, which decreases from around 800 to 600 ps as the solvent polarity is systematically increased, is further reduced to <200 ps in **C**. An overview of the steady‐state state absorption and fluorescence, as well as the TCSPC measurements in xylene is outlined in Table 1 (additional solvents are included in Tables S1–S4).

**Table 1:** Photophysical parameters of **B**, **A**, and **C** in xylene.^[a]^

Compound	λ_abs_/nm		λ_em_/nm		Φ_F_		τ_F_/ps	
	SubPz	Pnc_2_	SubPz	Pnc_2_	SubPz	Pnc_2_	SubPz	Pnc_2_
**A**	561.5	–	667	–	0.024	–	854	
**B**	–	661.5	–	669	–	0.025	–	<200^[b]^
**C**	563	661.5	–^[c]^	667.5	–	0.016	–	200^[b]^

[a] Values refer to the **A** centered fluorescence if not stated otherwise. [b] Lifetime is below the resolution limit of our time‐correlated single photon counting (TCSPC) setup. [c] **A** centered fluorescence is not detectable / overlapped by the **B** centered emission in the conjugate.

**Figure 3:**
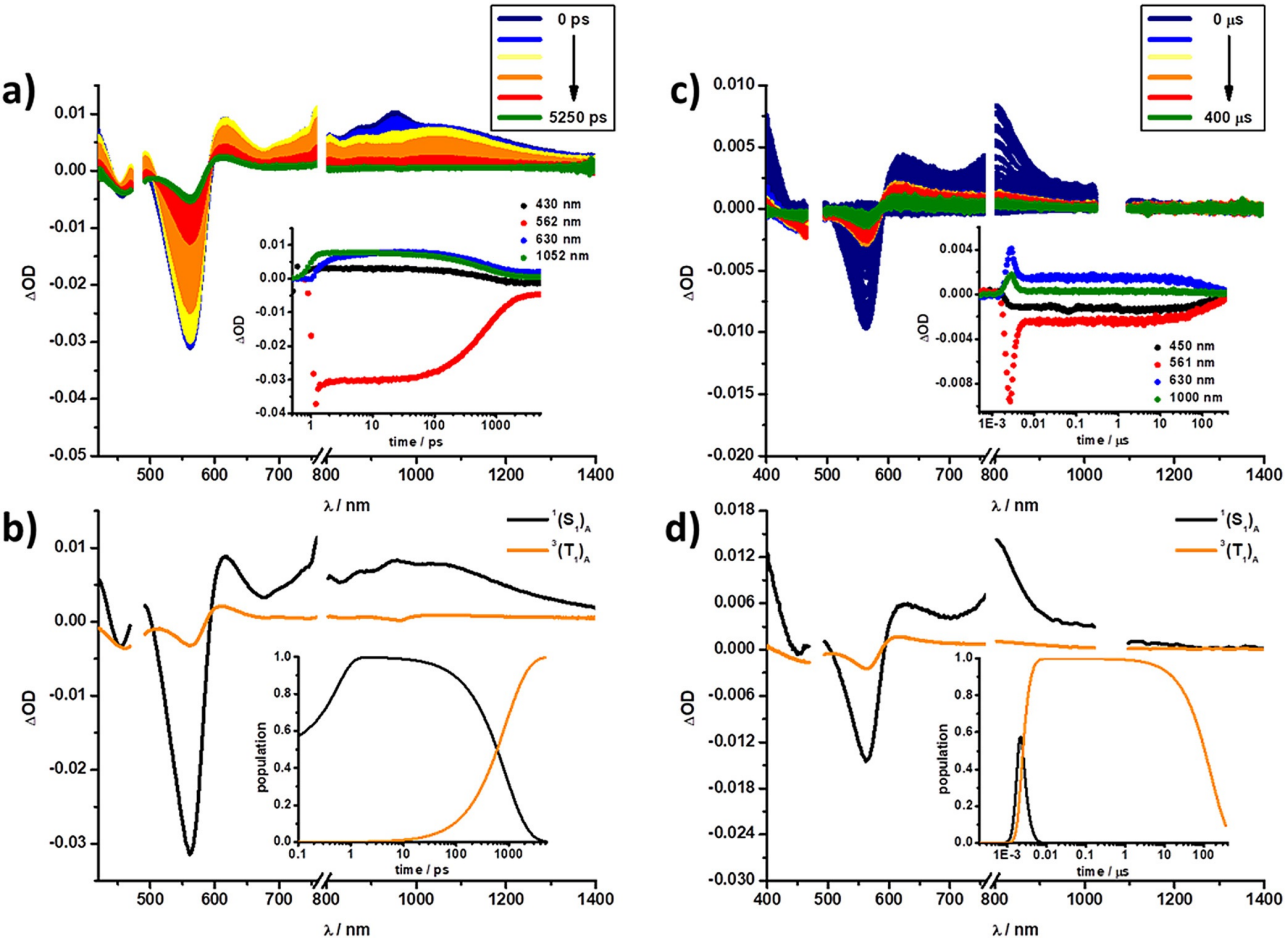
(a) Differential absorption spectra obtained from fsTA measurements (λ_ex_=480 nm) of **A** in xylene with several time delays between 0 and 5250 ps (colored solid lines) at rt; inset displays kinetics at 430, 562, 630, and 1052 nm (colored dots). (b) Species associated spectra (SAS) of the fsTA data of **A** shown in (a), with the initially formed singlet excited state ^1^(S_1_)_**A**_ (black) and the triplet excited state ^3^(T_1_)_**A**_ (orange); inset displays the relative populations of the SAS. (c) Differential absorption spectra obtained from nsTA measurements (λ_ex_=480 nm) of **A** in xylene with several time delays between 0 and 400 μs (colored solid lines) at rt; inset displays kinetics at 450, 561, 630, and 1000 nm (colored dots). (d) Species associated spectra (SAS) of the nsTA data of **A** shown in (c), with the initially formed singlet excited state ^1^(S_1_)_**A**_ (black) and the triplet excited state ^3^(T_1_)_**A**_ (orange); inset displays the relative populations of the respective SASs.

Transient absorption measurements on the femto‐ (fsTA) and nanosecond (nsTA) timescales were performed to corroborate deactivation kinetics of the investigated systems. In general, an excitation wavelength of 480 nm was selected for **A** (Figure 3) and **C**, while for **B** an excitation of 633 nm was chosen.

Starting with **A** in xylene (Figure 3), photoexcitation is followed by the immediate population of the singlet excited state ^1^(S_1_)_**A**_ including a narrow maximum at 615 nm and a broad maximum between 800 and 1200 nm, as well as ground state bleaching (GSB) at 561 nm. These features are subsequently replaced by those of the triplet excited state ^3^(T_1_)_**A**_, formed via slow intersystem crossing (ISC), which goes hand‐in‐hand with a shift of the maximum to 610 nm and the GSB to 564 nm. This two‐species kinetic model was applied for fitting the data in the other solvents, leading to similar transient absorption characteristics (Figures S28, S32 and S34).

For **B**, a kinetic model including three‐species was applied for all solvents, as previously developed.[^35^, ^36^] In short, the initially populated singlet excited state ^1^(S_1_S_0_)_**B**_ with maxima at 451, 510, and 1383 nm and GSB between 550 and 750 nm, transforms rapidly into the singlet correlated triplet pair state ^1^(T_1_T_1_)_**B**_, with maxima at 472, 506, 858, and 972 nm and GSB between 550 and 750 nm (in all solvents). The timescale on which this transition proceeds, <100 ps, is remarkable and clearly significantly faster than conventional ISC in pentacenes (10 ns).

**Figure 4:**
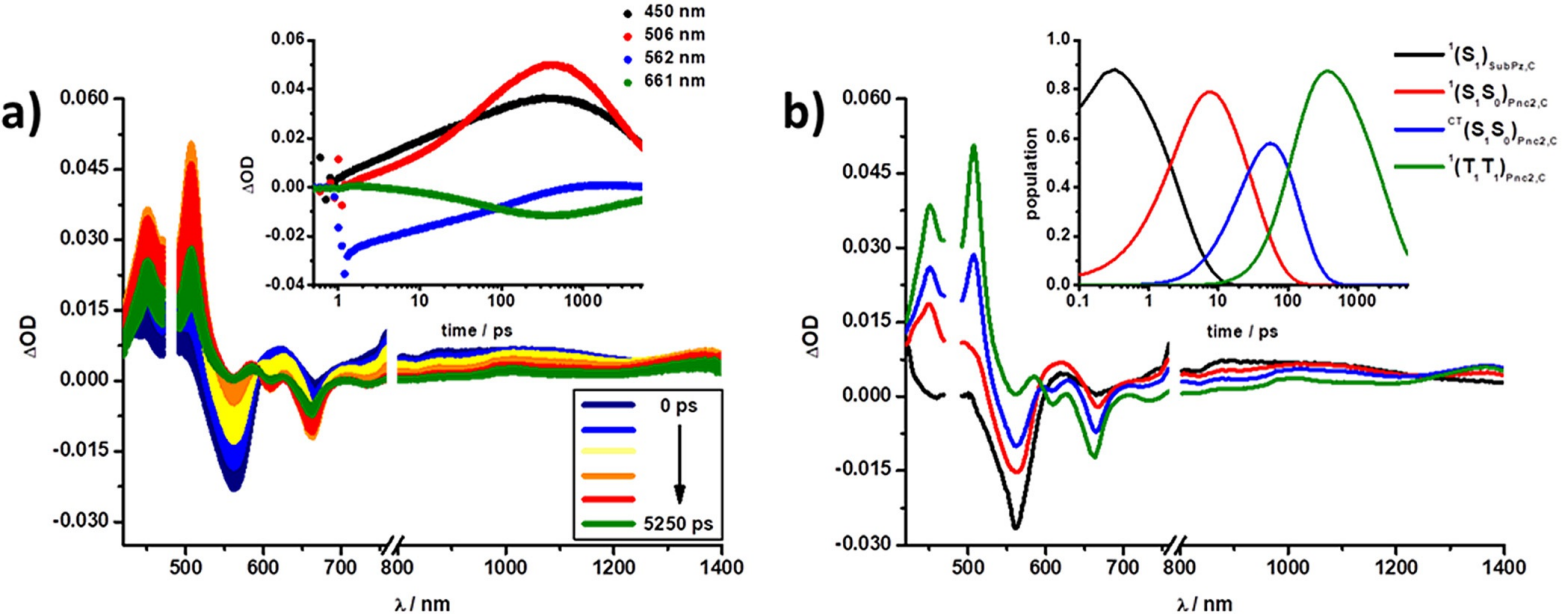
(a) Differential absorption spectra obtained from fsTA measurements (λ_ex_=480 nm) of **C** in xylene with several time delays between 0 and 5250 ps (colored solid lines) at rt; inset displays kinetics at 450, 506, 562, and 661 nm (colored dots). (b) Species associated spectra (SAS) of the fsTA data of **C** shown in (a), with the initially‐formed SubPz singlet excited state ^1^(S_1_)_**SubPz,C**_ (black), the Pnc_2_ singlet excited state ^1^(S_1_S_0_)_**Pnc2,C**_ (red), intermediate state ^CT^(S_1_S_0_)_**Pnc2,C**_ (blue), and correlated triplet pair state ^1^(T_1_T_1_)_**Pnc2,C**_ (green); inset displays the relative populations of the respective SAS.

Of great importance is the increased intensity of the GSB, which establishes the formation of more than a single triplet excited state per **B**. In other words, our findings confirm that the nature for the triplet excited state population is SF. A closer look at the fingerprints at 860, 970, and 1400 nm reveals that the correlated triplet pair ^1^(T_1_T_1_) is formed via an intermediate state that carries both singlet excited state and charge transfer (CT) character ^CT^(S_1_S_0_)_**B**_.[^35^, ^36^] In the last step, ^1^(T_1_T_1_)_**B**_ decays and leads to direct recovery of the ground state (S_0_S_0_) within a couple of nanoseconds. This is facilitated by strong coupling between the two pentacenes (both triplet as excited states), which activates geminate triplet‐triplet annihilation (Figures S27, S29, S31 and S35).^[44]^ Finally, for **C** a four‐species kinetic model is needed for analysis in all solvents, as characteristics from both **A** and **B** are present in the deactivation cascade. In xylene, the first observed species is the singlet excited state arising from the SubPz unit ^1^(S_1_)_**SubPz,C**_, with GSB at 561 nm and a broad maximum between 800 and 1200 nm. In contrast to the slow decay seen for the independent **A** molecule, the ^1^(S_1_)_**SubPz,C**_ state in **C** rapidly decays within ≈3 ps. Moreover, the ^1^(S_1_)_**SubPz,C**_ state is replaced by a transient, ^1^(S_1_S_0_)_**Pnc2,C**_, that resembles that of the singlet excited state of **B**, representing i‐FRET from SubPz to Pnc_2_. The subsequent steps follow the same deactivation pattern described for **B**, namely the population of the intermediate state ^CT^(S_1_S_0_)_**Pnc2,C**_, followed by the correlated triplet pair ^1^(T_1_T_1_)_**Pnc2,C**_ and lastly, the recovery of the ground state (S_0_S_0_) (Figure 4 and Figures S30, S33 and S36).

While the number of species and general deactivation mechanism for **C** remained consistent in all solvents, the choice of the solvent influences the efficiency of individual processes. At this point, we consider the redox chemistry of SubPz and Pnc_2_. Pnc_2_ oxidation and reduction occur at +0.44 and −1.43 eV, respectively, while those of SubPz are seen at +0.81 and −1.28 V, respectively—all vs. Fc/Fc^+^.[^30^, ^39^] Correspondingly, (S_1_)_**A**_ with 2.02 eV and (S_1_)_**B**_ with 1.82 eV, will only power the Pnc_2_ oxidation/SubPz reduction at 1.72 eV, but not the SubPz oxidation/ Pnc_2_ reduction at 2.24 eV. Despite exergonic driving forces of 0.3 and 0.1 eV, i‐FRET and i‐SF outcompete any excited state charge transfer due to good spectral overlap (*J*) and strong electronic couplings.

As the steady‐state assays demonstrated, the best spectral overlap between the fluorescence from the SubPz and the absorption of the Pnc_2_ groups is realized in the most apolar solvent xylene. Spectral overlap steadily decreases as solvent polarity increases from xylene to toluene, anisole, and benzonitrile. An immediate consequence of this trend is that the highest and lowest i‐FRET rate constants (*k*_FRET_) 3.52×10^11^ and 1.14×10^11^ s^−1^ are in xylene and benzonitrile, respectively.

Independent manifestation for the solvent dependence came from the lifetime of the ^1^(S_1_)_A_ state, which increases from 2.84 ps in xylene to 8.65 ps benzonitrile. The TQY follows a slightly different behavior and remains highest in xylene (171%±10 %) due to the fastest i‐FRET. It decreases to 152 % and 151%±10 % in toluene and anisole, respectively, in line with rates of the i‐FRET. In benzonitrile, however, the TQY increases again to 161%±10 %, despite the fact that the lowest k_FRET_ is found in benzonitrile. This is explained by the fact that i‐SF in Pnc_2_ proceeds via the intermediate CT state, ^CT^(S_1_S_0_)_**B**_; ergo a more polar solvent is beneficial. Overall, this showcases the vital need to balance the delicate relationship between efficient i‐FRET and i‐SF. The former is based on the optimal spectral overlap, while the latter proceeds via an intermediate CT state that is typically more efficient in polar solvents. This fact renders SubPzs a much more versatile energy donor than either ZnPzs or SubPcs.[^35^, ^36^] A summary of the kinetic data and triplet quantum yields in xylene is given in Table 2 (other solvents are included in Tables S1‐S4), while Table 3 shows a summary of the parameters used to calculate the i‐FRET rate constants for all solvents.

**Table 2:** Kinetic data from fsTA and nsTA measurements and triplet quantum yields (TQYs) for **A**, **B**, and **C** in xylene.

Compound	fsTA					nsTA	
	^1^(S_1_)SubPz	^1^(S_1_S_0_)Pnc_2_	CT(S_1_S_0_)Pnc_2_	^1^(T_1_T_1_)Pnc_2_	TQY^[a,b]^	^1^(S_1_)SubPz	^3^(T_1_)SubPz
**A**	863 ps	–	–	–	–	975 ps	152.01 μs
**B**	–	7.13 ps	74.93 ps	3.26 ns	132 %	–	–
**C**	2.84 ps	31.89 ps	97.14 ps	2.45 ns	171 %	n.r.^[c]^	–

[a] Determined via following the intensity of the transient bleaching of **B** and comparing the relative intensity of the species‐associated spectra for the ^CT^(S_1_S_0_)_Pnc2_ and the ^1^(T_1_T_1_)_Pnc2_ state, respectively. [b] An error margin in the range of ±10% may be considered when determining the TQYs. [c] n.r. = not resolvable. Lifetimes are either too long to be resolved with fsTA or too short to be resolved with nsTA.

**Table 3:** Parameters used to calculate the i‐FRET rate constants in xylene, toluene, anisole, and benzonitrile.

Solvent	τ_DA_/ps (TA)	τ_D_/ps (TCSPC)	*ϵ*_A_[a]/ M^−1^ cm^−1^	ϕ_F_D	J/M^−1^ cm^−1^ nm^4^	R_0_/A	R/A	K_FRET_/ 10^11^ s^−1^
Xylene	2.84	854	54 509	0.024	3.48×10^15^	31.39	12.13	3.52
Toluene	3.37	785	51 495	0.018	3.33×10^15^	29.80	12.02	2.96
Anisole	5.57	683	49 943	0.0051	2.87×10^15^	23.36	10.49	1.79
Benzonitrile	8.65	598	50 335	0.0020	2.29×10^15^	19.13	9.47	1.14

[a] For *ϵ*_A_, the extinction coefficients at the long‐wavelength maximum of the pentacene dimer (**B**) was taken.

## Conclusion

To summarize, a novel antennae‐bearing pentacene dimer, **C**, consisting of a bispentacene connected to the axial position of a hexakis‐(3,4,5‐trimethoxyphenyl)subporphyrazine through a carboxylic ester, has been successfully synthesized. This conjugate has been characterized using steady‐state absorption/fluorescence and time‐resolved transient absorption techniques. The unique tunability of the SubPz energy donor allowed adjustment of absorption and fluorescence to complement those of the bispentacene moiety as energy acceptor. Steady‐state absorption measurements showed a vastly improved panchromaticity due to the broad and strong absorptions of the SubPz, in comparison to previously reported **SubPcPnc_2_** and **ZnPzPnc_2_**.

**Figure 5:**
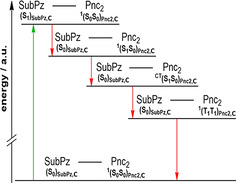
Qualitative energy diagram depicting the deactivation cascade in photoexcited **C**. The initial population of the SubPz singlet excited state (^1^(S_1_)_**SubPz,C**_) is followed by i‐FRET to yield the singlet excited state of Pnc_2_ (^1^(S_1_S_0_)_**Pnc2,C**_), subsequently performing i‐SF and producing the correlated triplet pair state ^1^(T_1_T_1_)_**Pnc2**_,_**C**_ via the intermediate state (^CT^(S_1_S_0_)_**Pnc2,C**_), before a TTA back to the **C** ground state concludes the deactivation process.

Steady‐state fluorescence measurements, in turn, revealed solvatochromic fluorescence of the SubPz, which afforded a red‐shifted emission of up to 20 nm in the most polar medium. This, in turn, drastically impacts the spectral overlap between the fluorescence of the SubPz and absorption of the Pnc_2_, which has major implications on the subsequent i‐FRET events. Time‐resolved transient absorption measurements verified efficient i‐FRET, that is, funneling singlet excited state energy from SubPz to Pnc_2_, populating the singlet excited state of Pnc_2_, and initiating i‐SF. This mechanism of i‐SF includes the population of an intermediate CT state that transitions to the correlated triplet pair. A qualitative depiction of the deactivation cascade is given in Figure 5.

Transient absorption measurements confirmed that solvent polarity can influence both i‐FRET and triplet quantum yields, with maxima in xylene of 3.52×10^11^ s^−1^ and 171%±10 %, respectively, as a result of the best spectral overlap. Interestingly, the second highest yield of 161%±10 % was found in benzonitrile, despite the lowest i‐FRET rate constant of 1.14×10^11^ s^−1^. This is rationalized by the fact that i‐SF of Pnc_2_ proceeds through a CT intermediate, which accelerates population of the correlated triplet pair. i‐SF is also enhanced in polar solvents, where the energy level of the CT state is stabilized, increasing the driving force of i‐SF. A three‐fold increase in the rate of FRET in xylene, compared to benzonitrile, offers a straightforward route to circumvent the established challenge that i‐SF is typically unfavorable in nonpolar solvents (which increase the energy of a CT state).

Overall, with the help of a SubPz, a broadened and more intense absorption was introduced in the 400 to 600 nm range of **C** relative to the **SubPcPnc_2_** and **ZnPzPnc_2_** conjugates. Of even greater value is the shift in fluorescence, that emerges in the most polar solvents, and the accompanying influence on *J* and *k*_FRET_, which offers a means to tune the cascade of i‐FRET and i‐SF without synthetic modification of the molecular structure.

Electronic Supplementary Information (ESI) available: [Detailed synthetic procedures and complete spectroscopic characterization (^1^H and ^13^C NMR, FTIR, UV/Vis, MS and HRMS spectra) and photophysical characterization (steady‐state absorption and fluorescence, time‐correlated single photon counting (TCSPC), femto‐ (fsTA) and nanosecond (nsTA) transient absorption spectroscopy, triplet quantum yield (TQY) determination, FRET rate constant determination]

## Conflict of interest

The authors declare no conflict of interest.

## Supporting information

As a service to our authors and readers, this journal provides supporting information supplied by the authors. Such materials are peer reviewed and may be re‐organized for online delivery, but are not copy‐edited or typeset. Technical support issues arising from supporting information (other than missing files) should be addressed to the authors.

Supplementary
