# Supplementary material for: Controlling Intramolecular Förster Resonance Energy Transfer and Singlet Fission in a Subporphyrazine–Pentacene Conjugate by Solvent Polarity
Source: Angew Chem Int Ed Engl. 2020 Nov 18;60(3):1474–81. doi: 10.1002/anie.202011197 (PMC7839765; doi:10.1002/anie.202011197)
Supplement: Supplementary file 1 — Supplementary [file ANIE-60-1474-s001.pdf]

Angewandte Chemie
Eine Zeitschrift der Gesellschaft Deutscher Chemiker

Supporting Information

Controlling Intramolecular Förster Resonance Energy Transfer and Singlet Fission in a Subporphyrazine–Pentacene Conjugate by Solvent Polarity

David Guzmán[+], Ilias Papadopoulos[+], Giulia Lavarda, Parisa R. Rami, Rik R. Tykwinski, M. Salomé Rodríguez-Morgade,* Dirk M. Guldi,* and Tomás Torres**

anie_202011197_sm_miscellaneous_information.pdf

Table of Contents

General Procedures, Materials, and Methods	S3–S6
Synthesis and Characterization	S7–S33
Photophysics	S34–S51
References	S52

General Procedures, Materials and Methods

Synthesis and characterization

Abbreviations (*Synthesis and characterization*). CuTC = copper (I) thiophene-2-carboxylate; DCM = dichloromethane; DCTB = *trans*-2-[3-(4-*tert*butylphenyl)-2-methyl-2-propenylidene] malononitrile; DMAP = 4-dimethylaminopyridine; DMI = 1,3-dimethyl-2-imidazolidinone; EDC = 1-ethyl-3-(3-dimethylaminopropyl)carbodiimide; EtOAc = ethyl acetate; FT-IR = Fourier-transform infrared spectroscopy; HRMS = high resolution mass spectrometry; MALDI-TOF = matrix-assisted laser desorption/ionization - time of flight; MS = mass spectrometry; NMR = nuclear magnetic resonance; Pnc_2 = pentacene dimer; PPG = polypropylene glycol; ppm = part per million; SubPz = subporphyrazine; TLC = thin layer chromatography.

Syntheses and Purifications. All manipulations were carried out under an argon atmosphere using conventional Schlenk techniques. **Pnc_2COOH**,[1] bis(propylthio)maleonitrile,[2] and CuTC[3] were prepared following reported procedures. The rest of chemicals were purchased from commercial suppliers and used without further purification. “Synthetic grade” solvents were used for chemical reactions, column chromatography purifications, and “anhydrous grade” for reactions under dry conditions. Additionally, some solvents were further dried by distillation with sodium/benzophenone [tetrahydrofuran (THF)] or with a solvent purifying system by Innovative Technology Inc. MD-4-PS. Solid, hygroscopic reagents were dried in a vacuum oven before use. Column chromatography was carried out on silica gel Merck-60 (230-400 mesh, 60 Å) and TLC was performed on aluminum sheets pre-coated with silica gel 60 F_{254} (Merck). Size exclusion chromatography was performed on Bio-Beads^{TM} S-X1 Support (200-400 mesh).

Instrumental Analyses. ^{1}H NMR and ^{13}C NMR spectra were obtained using a Bruker Advance 400 (400 MHz) spectrometer. The temperature was actively controlled at 298 K, unless indicated otherwise. Chemical shifts are measured in ppm relative to tetramethylsilane (TMS). Matrix-assisted laser desorption/ionization time of flight (MALDI-TOF) MS and high-resolution mass spectrometry (HRMS) spectra were recorded with a BRUKER ULTRAFLEX III instrument equipped with a nitrogen laser operating at 337 nm. A convenient matrix of DCTB was used for the measurements of the SubPz derivatives. Infrared (FT-IR) spectra were recorded on a

Bruker Alpha II spectrometer by attenuated total reflection (ATR) technic, in all cases solid samples. Previous UV/Vis (λ_{max} and ε) and fluorescence spectra were recorded with a JASCO V-660-spectrophotometer and with a JASCO V8600-spectrofluorometer, respectively.

Photophysics

Steady-State Absorption and Fluorescence. Steady-state absorption experiments were performed on a Perkin Elmer Lambda 2 spectrometer at room temperature (rt). Steady-state fluorescence experiments were carried out on a FluoroMax3 spectrometer from Horiba in the visible detection range at rt. The data was recorded using Fluorescence software from Horiba Jobin Yvon. The fluorescence quantum yields (Φ_F) were obtained by means of gradient analyses using Nile blue a (Φ_F = 0.27) in methanol for the SubPz centered fluorescence and zinc phthalocyanine (Φ_F = 0.30) in toluene for the Pnc_2 centered fluorescence. All measurements were performed in 10 x 10 mm quartz cuvettes at rt.

Time-Correlated Single Photon Counting (TCSPC). Fluorescence lifetimes were recorded *via* the TCPSC setup using a FluoroLog3 emission spectrometer, from Horiba JobinYvon, equipped with an R3809U-58 MCP, from Hamamatsu, and an EXW-6 (NKT) SuperK Extreme high power supercontinuum fiber laser with excitation wavelengths at 480 and 633 nm (~150 ps fwhm) for the SubPz and Pnc_2 centered fluorescence, respectively.

Femto- (fsTA) and Nanosecond (nsTA) Transient Absorption Spectroscopy. FsTA (0 to 5250 ps) and nsTA (1 ns to 400 µs) measurements were carried out with an amplified Ti:sapphire CPA-2110 fs laser system (Clark MXR: output 775 nm, 1 kHz, 150 fs pulse width) using the HELIOS and EOS transient absorption pump/probe detection systems from Ultrafast Systems. Solutions were purged with argon for ~15 minutes in 2 x 10 mm cuvettes. The 480 and 633 nm excitation wavelengths, with respective energies of 400 nJ, were generated *via* a noncolinear optical parametric amplifier from Clark MXR (NOPA). TA spectra were obtained by the same HELIOS and EOS detection systems. In order to ensure a homogenous excitation of the probed area, the spot diameter of the pump beam was kept always larger than that of the probe beam. The delays of the probe pulses were controlled by a delay stage, for fsTA, or electronic response, for nsTA, with respect to the pump pulses, respectively. A beam splitter split the probe pulses into a signal and reference beam, before passing the sample. In order

to correct for fluctuations in the probe beam intensity, the probe and reference beam were detected independently. Furthermore the spectra were acquired shot-by-shot and averaged at each delay over ~1500 times, to ensure a reasonable signal-to-noise ratio.

FsTA and NsTA Data Evaluation. Results from fsTa and nsTA data have been analyzed *via* multiwavelength and GloTarAn target analysis. Target analysis was performed on the TA data sets using the proposed sequential model. The analytical solution to the coupled differential equations that describe the kinetic model is convoluted with a Gaussian instrument response function. After the least-squares fitting has converged, the raw data matrix is deconvoluted using the specific solution to the kinetic model and parameters from the fit to obtain the species-associated spectra (SAS) and their populations as a function of time. GloTarAn is a Java-based graphical user interface to the R-package TIMP, which was developed for global and target analysis of time-resolved spectroscopy data. The dispersion (chirp of the white-light pulse) of the instrument response function (IRF) was modeled, *via* Surface Xplorer by Ultrafast Systems, and taken into account during the fitting procedure.

Triplet Quantum Yield (TQY) Determination. The determination of the TQY followed the same procedure as in our previous works.[4,5] In brief summary, the TQY was obtained by following the intensification of the ground state bleach (GSB) relating to Pnc_2. The SAS were corrected, in order to account for residual SubPz centered contributions (e.g. GSB, fluorescence) of the Pnc_2 centered GSB. Equation (1) was used for the TQY calculation of the **Pnc_2COOH** reference compound:

$$\frac{\Delta OD_{SAS}(\ ^1(T_1T_1)_{Pnc_2}}{\Delta OD_{SAS}(\ ^1(S_1S_0)_{Pnc_2}}\ x\ 100\% = TQY \quad \textbf{(1)}$$

While equation (2) was used for the TQY calculation of the **SubPzPnc_2** conjugate:

$$\frac{\Delta OD_{SAS}(\ ^1(T_1T_1)_{Pnc_2}}{\Delta OD_{SAS}(^{CT}(S_1S_0)_{Pnc_2}}\ x\ 100\% = TQY \quad \textbf{(2)}$$

Here $\Delta OD_{SAS}(^{CT}(S_1S_0)_{Pnc2})$ rather than $\Delta OD_{SAS}(^1(S_1S_0)_{Pnc2})$ was utilized, as the singlet excited state of **SubPzOH** ($^1(S_1)_{SubPzOH}$) featured a positive transient in the area of the GSB of Pnc_2, therefore distorting the intensity of the GSB relating to $^1(S_1S_0)_{Pnc2}$.

FRET Rate Constant Determination. The determination of the FRET rate also followed the same procedure from previous works,[4],[5] using the following equations (3)-(7):

$$R = R_0 \left(\frac{1-E}{E}\right)^{\frac{1}{6}} \quad \textbf{(3)}$$

R = FRET distance; R_0 = critical energy donor-acceptor distance; E = FRET efficiency.

$$E = 1 - \frac{\tau_{DA}}{\tau_D} \quad \textbf{(4)}$$

E = FRET efficiency; τ_{DA} = experimental lifetime (TA) of energy donor-acceptor conjugate; τ_D = experimental fluorescence lifetime of energy donor (TCSPC).

$$R_0 = 0.2108\ (\kappa^2 \Phi_F n^{-4} J)^{\frac{1}{6}} \quad \textbf{(5)}$$

R_0 = critical energy donor-acceptor distance; κ^2 = orientation factor; n = refractive index of the solvent; *J* = degree of spectral overlap.

$$J = \int_0^\infty F_D(\lambda)\varepsilon_A(\lambda)\lambda^4 d\lambda \quad \textbf{(6)}$$

J = degree of spectral overlap; F_D = fluorescence intensity of the donor at wavelength λ.

The fluorescence spectra have to be normalized to fulfill equation (7); ε_A = extinction coefficient of the acceptor at wavelength λ

$$\int_0^\infty F_D(\lambda) d\lambda = 1 \quad \textbf{(7)}$$

The detailed procedure (including a description of a software-based algorithm to calculate the spectral overlap integral *J*) is described by Hink et al.[6] For κ^2 we used 2/3 (~0.67),[7] as often used for non-rigid systems ("dynamic isotropic limit").[8] The refractive indices n were taken as 1.506,[9] 1.496,[10] 1.518,[11] and 1.529[9] for xylene, toluene, anisole, and benzonitrile, respectively. See Table 3 for a summary of the parameters used for the FRET rate constant calculation as well as for an overview of the obtained values for the FRET constants.

Synthesis and Characterization

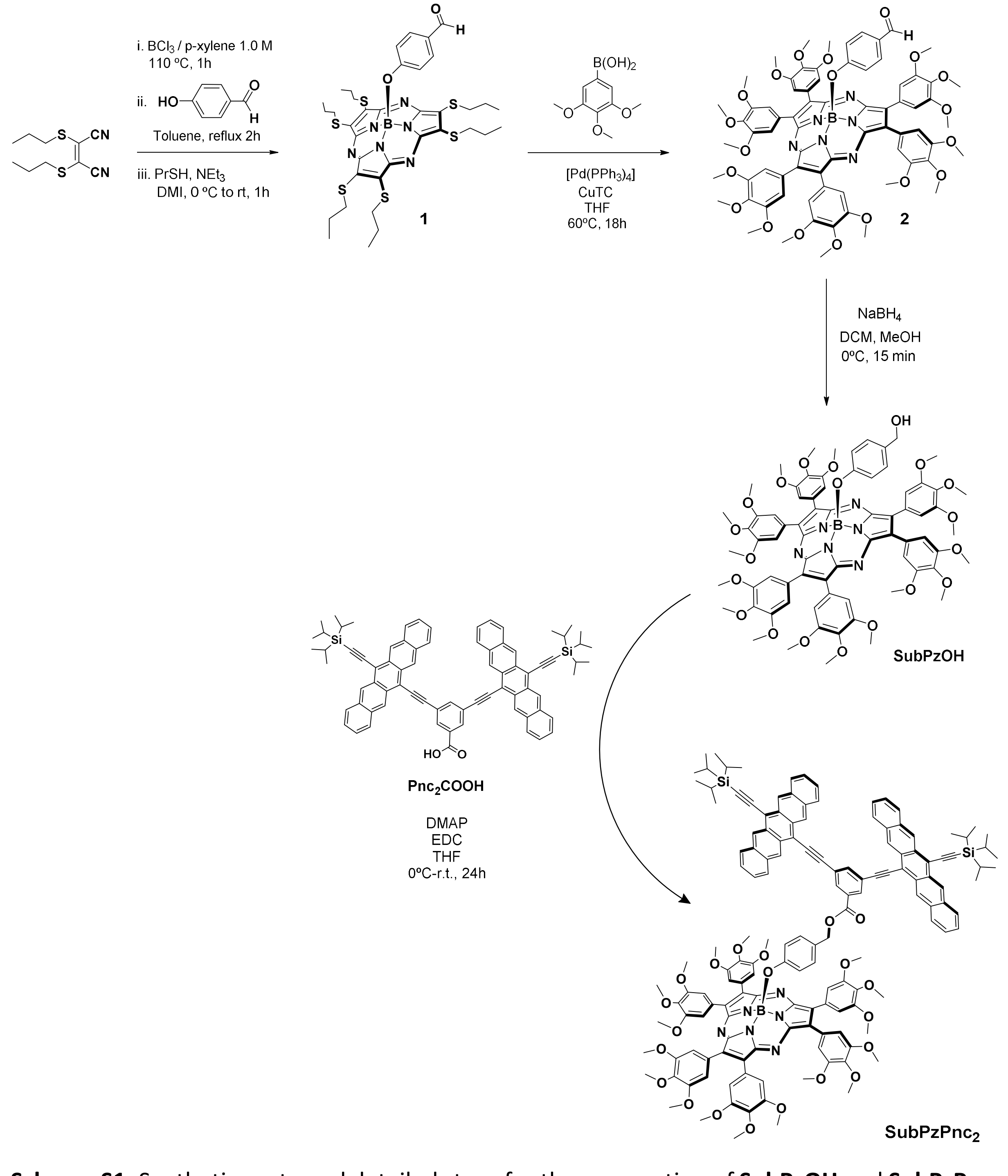

Scheme S1. Synthetic route and detailed steps for the preparation of **SubPzOH** and **SubPzPnc₂** conjugate.

4-Formylphenoxy[2,3,7,8,12,13-hexa(propylthio)subporphyrazinato]boron(III) (1)

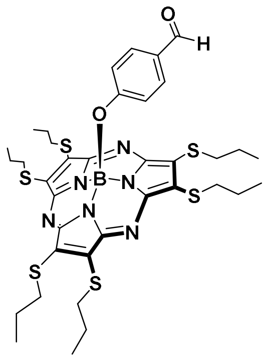

An oven-dried two-neck round-bottom flask connected to a condenser and held under an argon atmosphere was charged with bis(propylthio)maleonitrile (452.0 mg, 2.00 mmol). A 1.0 M solution of BCl_3 in p-xylene (2.0 mL, 2.00 mmol) was then added into the flask *via* a syringe, and the mixture was heated at 110 °C for 1 h. The reaction mixture was cooled to room temperature, and the solvent was removed under reduced pressure. The resulting dark violet crude was dissolved in anhydrous toluene (4 mL) and 4-hydroxybenzaldehyde (1.50 g, 12.30 mmol) was added. The mixture was stirred to reflux for 2 h under an argon atmosphere followed by removal of the solvent under reduced pressure once more. The black crude material was passed through a short pad of compressed Celite using a 9:1 mixture of n-hexane/ethyl acetate, followed by the column chromatography on silica gel (20:1 mixture of n-hexane/ethyl acetate), collecting the two red fractions close to each other. The red viscous solid obtained was dissolved in 25 mL of anhydrous DMI at 0 °C under an argon atmosphere. Then, 1-propanethiol (36 µL, 0.40 mmol, 0.2 equiv respect to bis(propylthio)maleonitrile) and freshly distilled triethylamine (56 µL, 0.40 mmol, 0.2 equiv respect to bis(propylthio)maleonitrile) were added *via* the syringe, and the resulting mixture was stirred heating from 0 °C to room temperature for 1 h. Once the reaction is completed (TLC showed just one red spot in n-hexane/ethyl acetate 20:1) the product was extracted with diethyl ether and washed three times with water, dried over $MgSO_4$, and the solvent was evaporated under reduced pressure. The crude product was chromatographed on silica gel (7:3 mixture of n-hexane/ethyl acetate) giving pure target compound **1** (65.0 mg, 12%) as a red viscous solid.

^{1}H NMR (400 MHz, $CDCl_3$): δ(ppm) = 9.69 (s, 1H), 7.37 (d, *J* = 8.0 Hz, 2H), 5.43 (d, *J* = 8.0 Hz, 2H), 4.08–4.01 (m, 6H), 3.65–3.58 (m, 6H), 2.05–1.87 (m, 12H), 1.17 (t, *J* = 7.2 Hz, 18H).

^{13}C NMR (100.6 MHz, $CDCl_3$): δ(ppm) = 190.6, 158.8, 154.1, 132.0, 131.1, 130.2, 119.4, 36.9, 23.7, 13.5.

FT-IR (ATR): ν (cm^{-1}) = 2961, 2929, 2870, 2727, 1695, 1597, 1573, 1477, 1450, 1287, 1235, 1172, 1119, 1001 (S), 895, 838, 749, 579.

UV/Vis ($CHCl_3$) λ_{max} (nm) (log ε/dm^3 mol^{-1} cm^{-1}): 280 (4.7), 442 (4.6), 558 (4.5).

Fluorescence (CHCl3): λ_{ex} = 525 nm; λ_{em} = 680 nm.

MS (MALDI-TOF, DCTB, +): *m/z* = 810.2 [M]$^+$; 689.1 [M – Axial ligand]$^+$

HRMS (MALDI-TOF): Calculated for [M]$^+$ $C_{37}H_{47}BN_6O_2S_6$ 809.2209; found 809.2253.

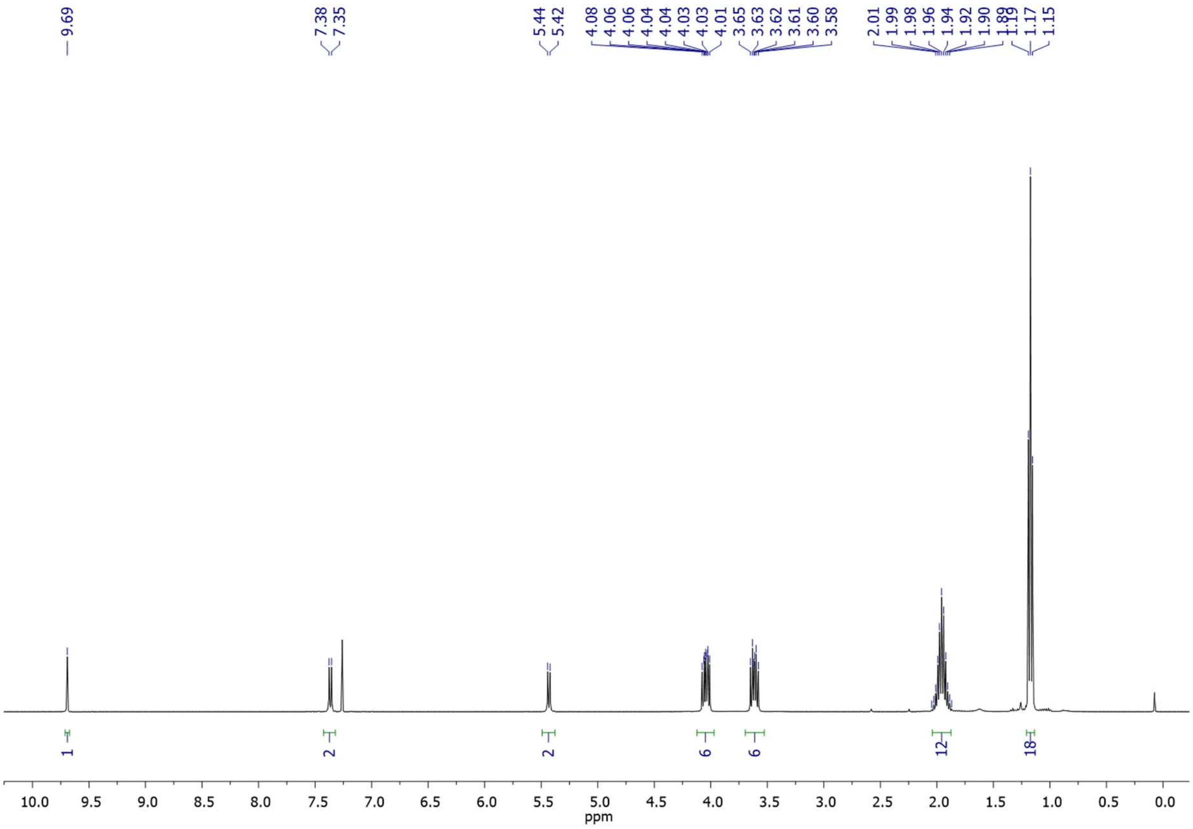

Figure S1. ^{1}H NMR (400 MHz) spectrum of **1** in $CDCl_3$ at 298 K.

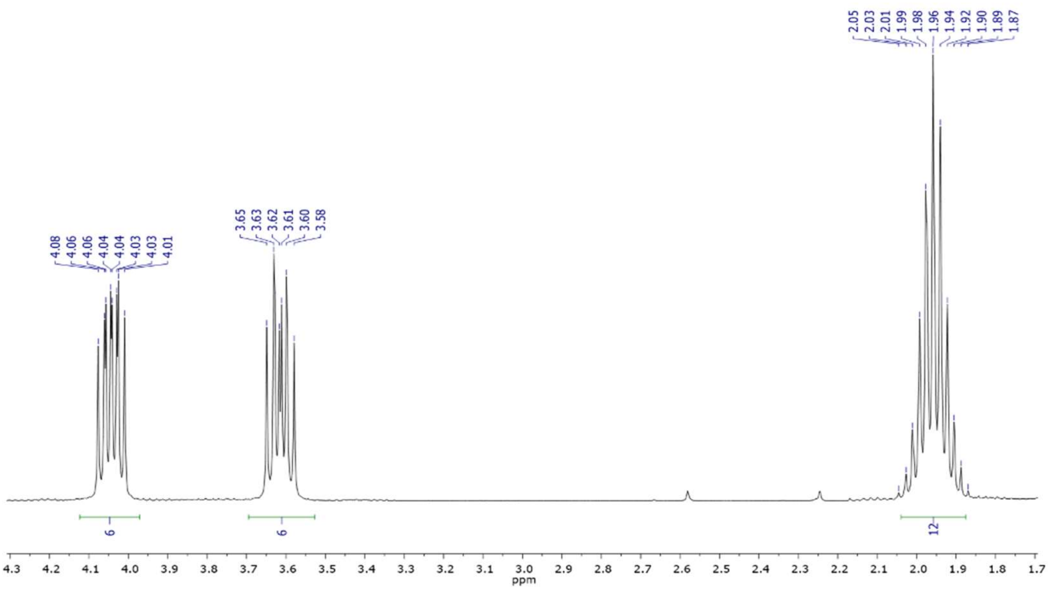

Figure S2. ^{1}H NMR spectrum of **1**, detail of the region between 4.3 ppm and 1.7 ppm.

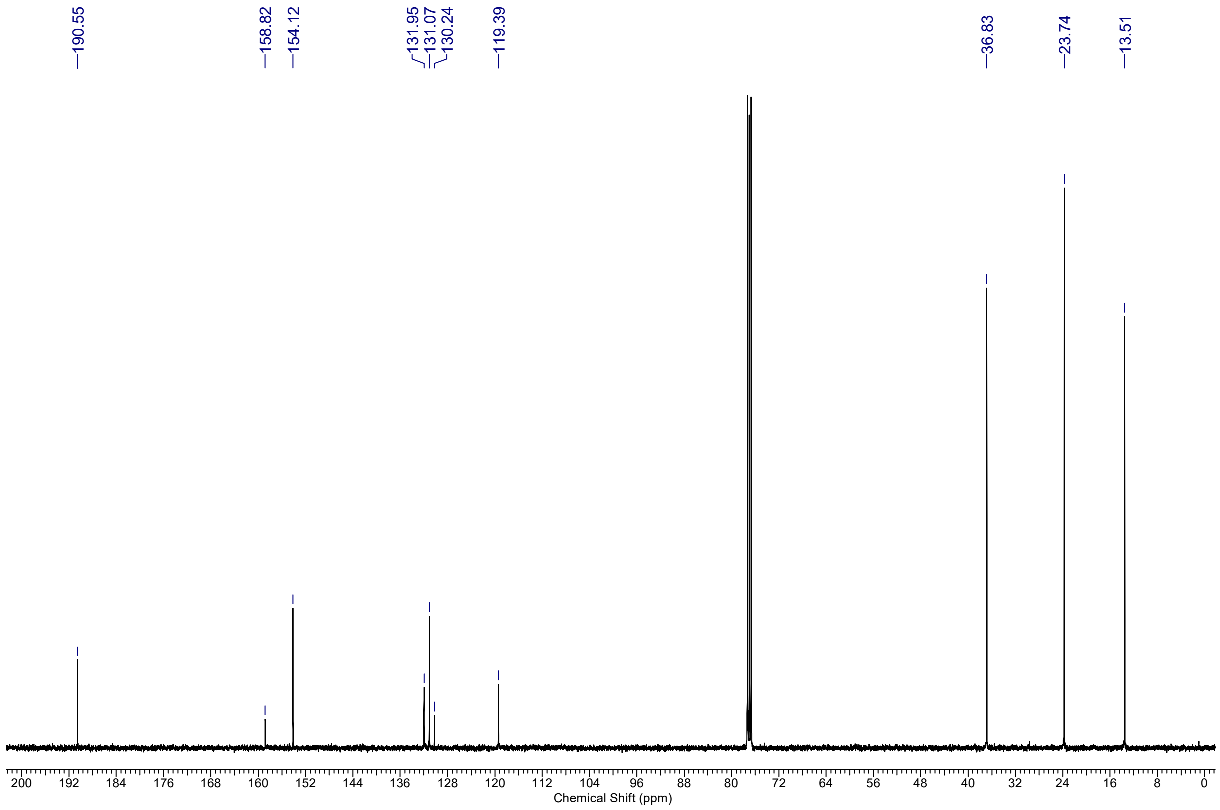

Figure S3. ^{13}C NMR (100.6 MHz) spectrum of **1** in $CDCl_3$ at 298 K.

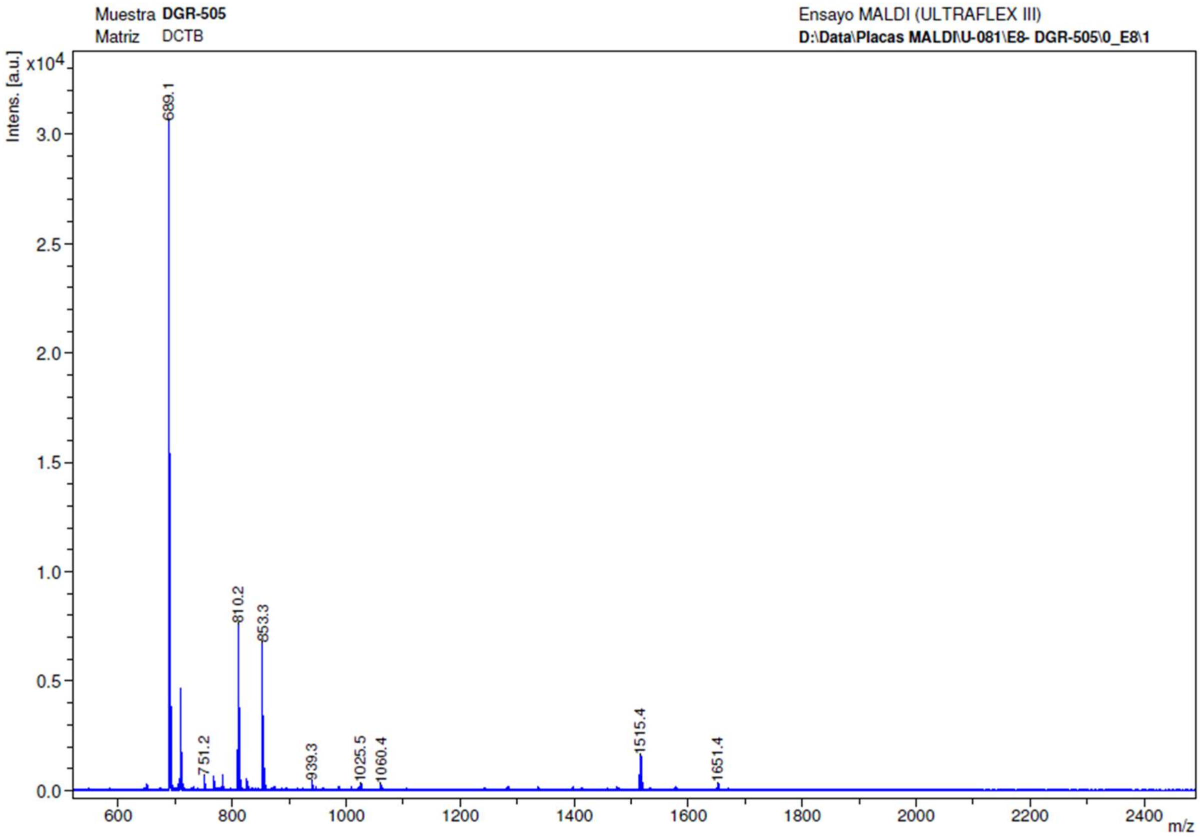

Figure S4. MALDI-TOF mass spectrum (in DCTB matrix) of **1**.

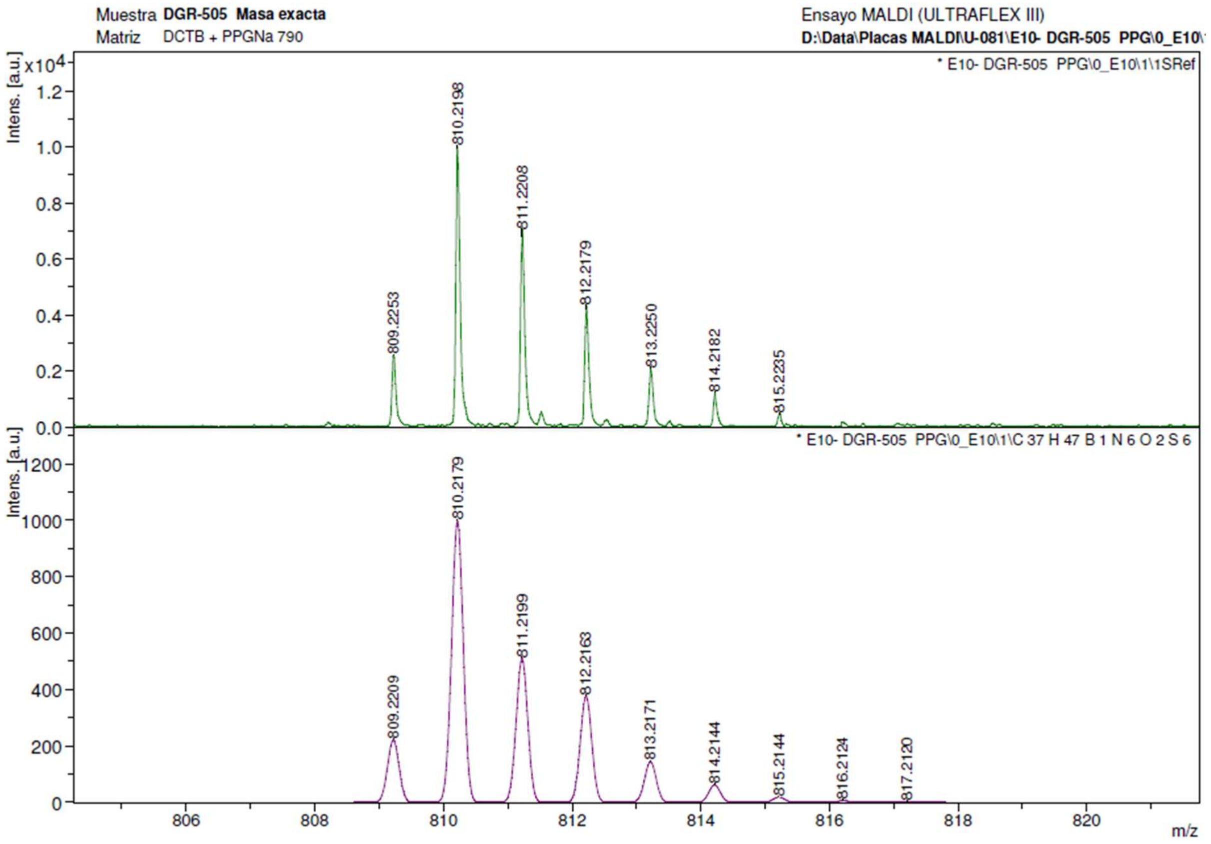

Figure S5. *Upper part* – HRMS (MALDI-TOF) spectrum (in DCTB+PPGNa 790 matrix) of **1**. *Lower part* – Calculated isotopic pattern of **1**.

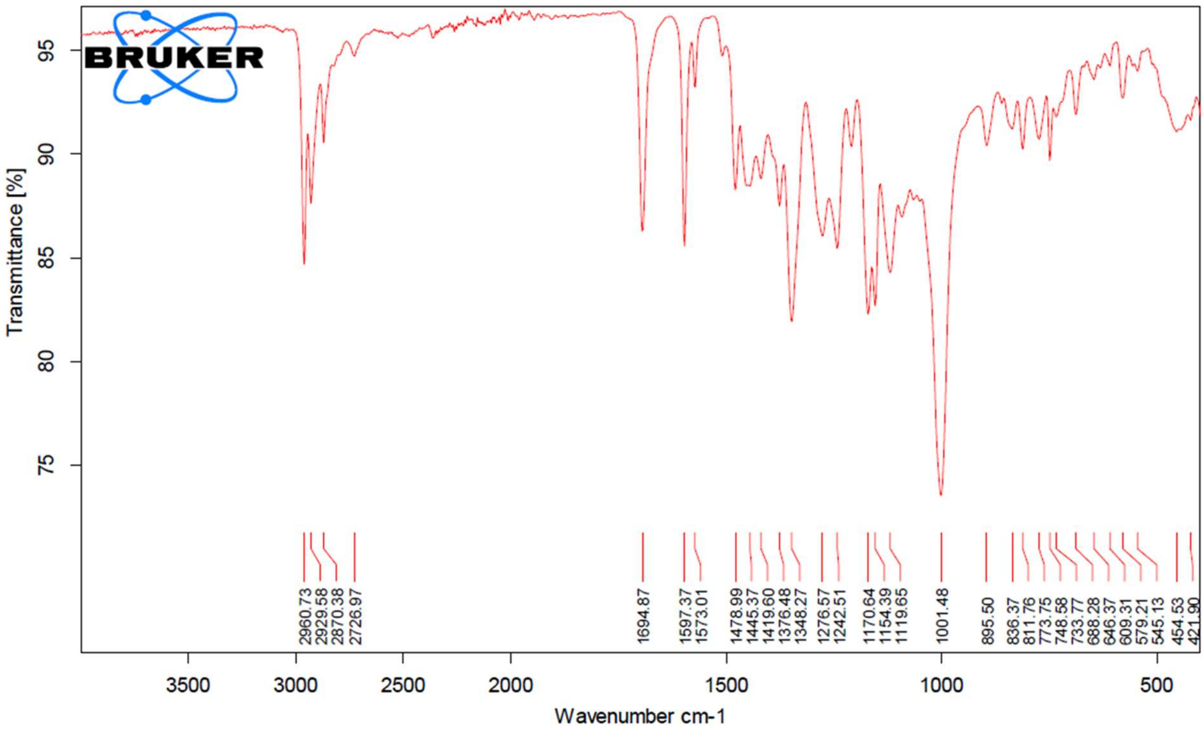

Figure S6. FT-IR spectrum (ATR) of **1**.

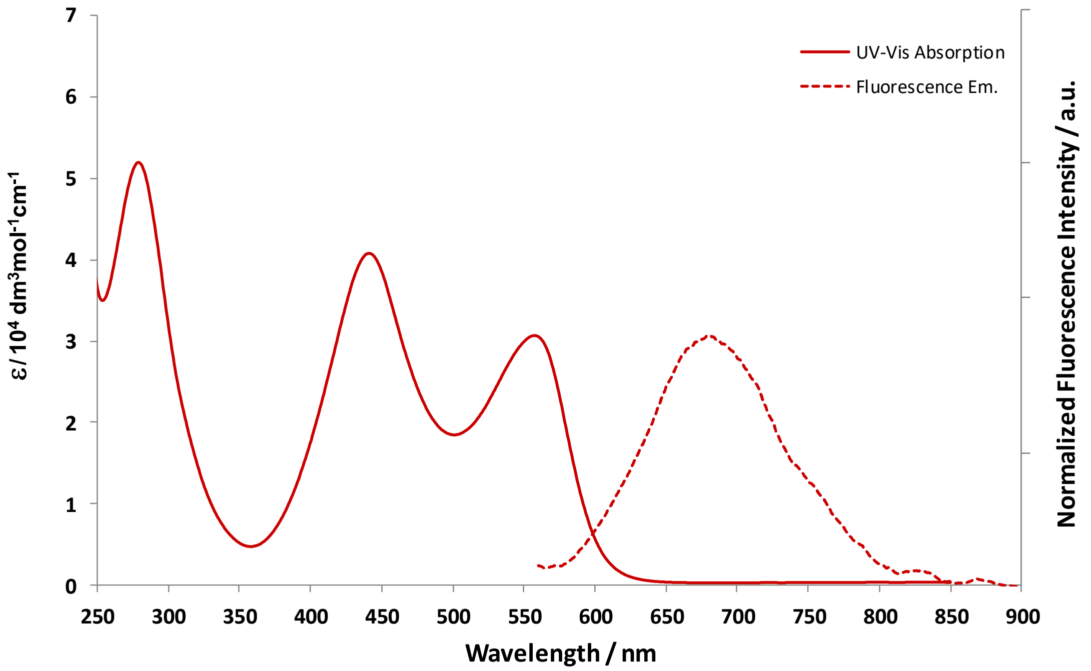

Figure S7. UV/Vis absorption (solid line) and normalized fluorescence (dashed line) spectra of **1** in chloroform.

4-Formylphenoxy[2,3,7,8,12,13-hexa(3'',4'',5''-trimethoxyphenyl)) subporphyrazinato]boron(III) (2)

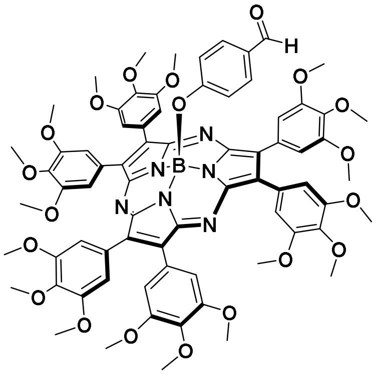

An oven-dried 10 mL Schlenk was over-dried and purged with argon during 10 min. Subsequently, hexa(propylthio)-subporphyrazine **1** (47.0 mg, 58.95 µmol), 3,4,5-trimethoxyphenylboronic acid (221.0 mg, 1.04 mmol), [Pd(PPh_3)$_4$] (40.9 mg, 35.37 µmol, 60 mol %), and CuTC (198.0 mg, 1.04 mmol) were added into the Schlenk and purged with argon for 10 min, followed by the addition of anhydrous THF (5 mL) *via* cannula. The reaction mixture was stirred at 60 ºC for 18 h under an argon atmosphere. Then, the mixture was cooled to room temperature and passed through a short silica gel column using THF as eluent. The solvent was removed and the residue was purified by silica gel column chromatography (mixture of n-hexane/ethyl acetate/THF 4 : 5.6 :0.4). The product **2** was obtained as a purple solid (28.5 mg, 36%).

^{1}H NMR (400 MHz, $CDCl_3$): δ(ppm) = 9.68 (s, 1H), 7.43 (d, *J* = 8.0 Hz, 2H), 7.15 (s, 12H), 5.55 (d, *J* = 8.0 Hz, 2H), 3.92 (s, 18H), 3.62 (s, 36H).

^{13}C NMR (100.6 MHz, $CDCl_3$): δ(ppm) = 190.6, 159.1, 156.8, 153.2, 138.7, 133.3, 132.0, 131.9, 131.5, 129.8, 128.5, 128.4, 126.7, 118.4, 108.6, 61.0, 56.0.

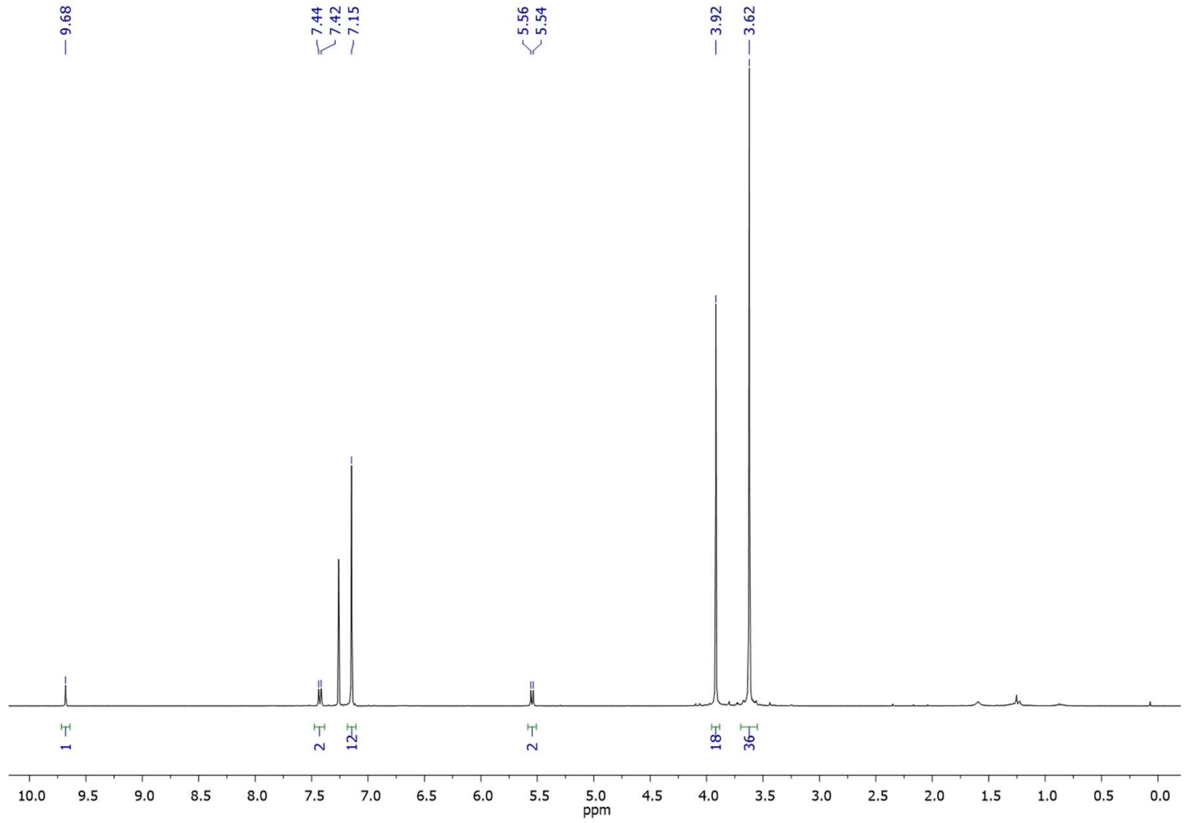

Figure S8. ^{1}H NMR (400 MHz) spectrum of **2** in $CDCl_3$ at 298 K.

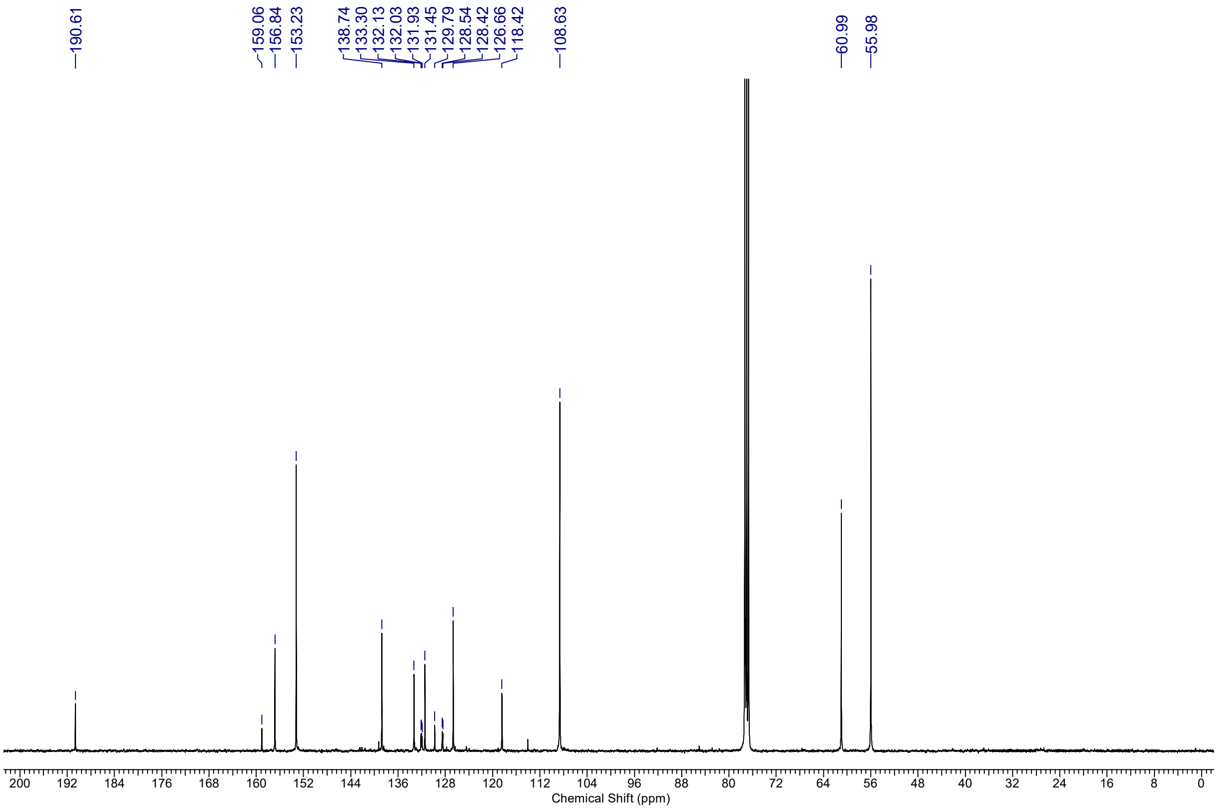

Figure S9. ^{13}C NMR (100.6 MHz) spectrum of **2** in $CDCl_3$ at 298 K.

4-(Hydroxymethyl)phenoxy[2,3,7,8,12,13-hexa(3'',4'',5''-trimethoxyphenyl))subporphyrazinato]boron(III) (SubPzOH)

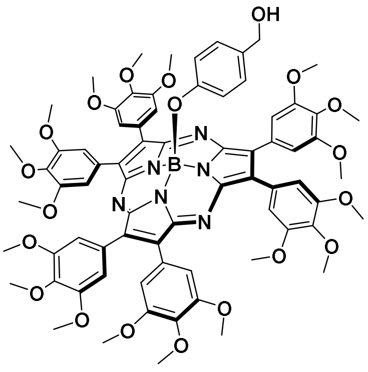

In an oven-dried 5 mL round-bottom Schlenk was charged with formyl-SubPz **2** (17.8 mg, 13.06 μmol), $NaBH_4$ (1.0 mg, 26.12 μmol) and anhydrous DCM (3 mL) under argon atmosphere. The solution was stirred at 0 ºC for 5 min in the dark, then MeOH (1.5 mL) was added dropwise. The resulting reaction mixture was stirred for 15 min at 0 ºC, and quenched by the addition of water (5 mL). The product was extracted with ethyl acetate, washed with brine, and dried over anhydrous $MgSO_4$. After filtration and evaporation, the solid residue was purified by silica gel column chromatography (mixture of n-hexane/ethyl acetate/THF 4 : 5.6 : 0.4) giving pure compound **SubPzOH** (13.5 mg, 76%) as a garnet solid.

^{1}H NMR (400 MHz, $CDCl_3$): δ(ppm) = 7.14 (s, 12H), 6.88 (d, *J* = 8.0 Hz, 2H), 5.50 (d, *J* = 8.0 Hz, 2H), 4.42 (s, 2H), 3.91 (s, 18H), 3.62 (s, 36H).

^{13}C NMR (100.6 MHz, $CDCl_3$): δ(ppm) = 156.8, 153.2, 152.7, 138.6, 133.3, 133.1, 128.2, 126.9, 118.5, 108.6, 64.8, 61.0, 56.0.

FT-IR (ATR): ν (cm^{-1}) = 3529, 2998, 2926, 2853, 1738, 1672, 1579, 1510, 1477, 1450, 1287, 1235, 1125 (S), 1008, 923, 870, 843, 717.

UV/Vis ($CHCl_3$) λ_{max} (nm) (log ε/dm^3 mol^{-1} cm^{-1}): 282 (4.8), 446 (4.6), 557 (4.7).

Fluorescence (CHCl3): λ_{ex} = 500 nm; λ_{em} = 672 nm.

MS (MALDI-TOF, DCTB, +): *m/z* = 1365.5 [M]$^+$; 1241.5 [M – Axial ligand]$^+$

HRMS (MALDI-TOF): Calculated for [M]$^+$ $C_{73}H_{73}BN_6O_{20}$ 1363.5004; found 1363.4943.

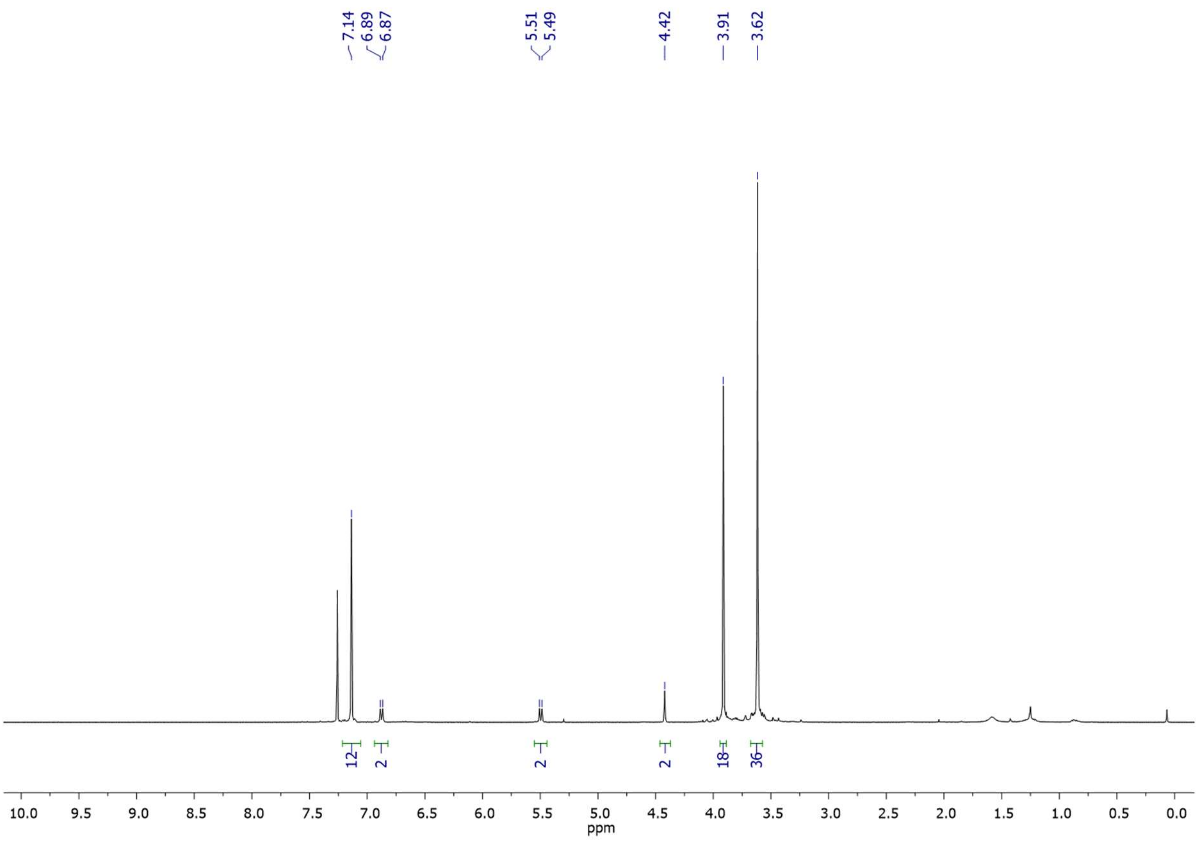

Figure S10. ^{1}H NMR (400 MHz) spectrum of **SubPzOH** in $CDCl_3$ at 298 K.

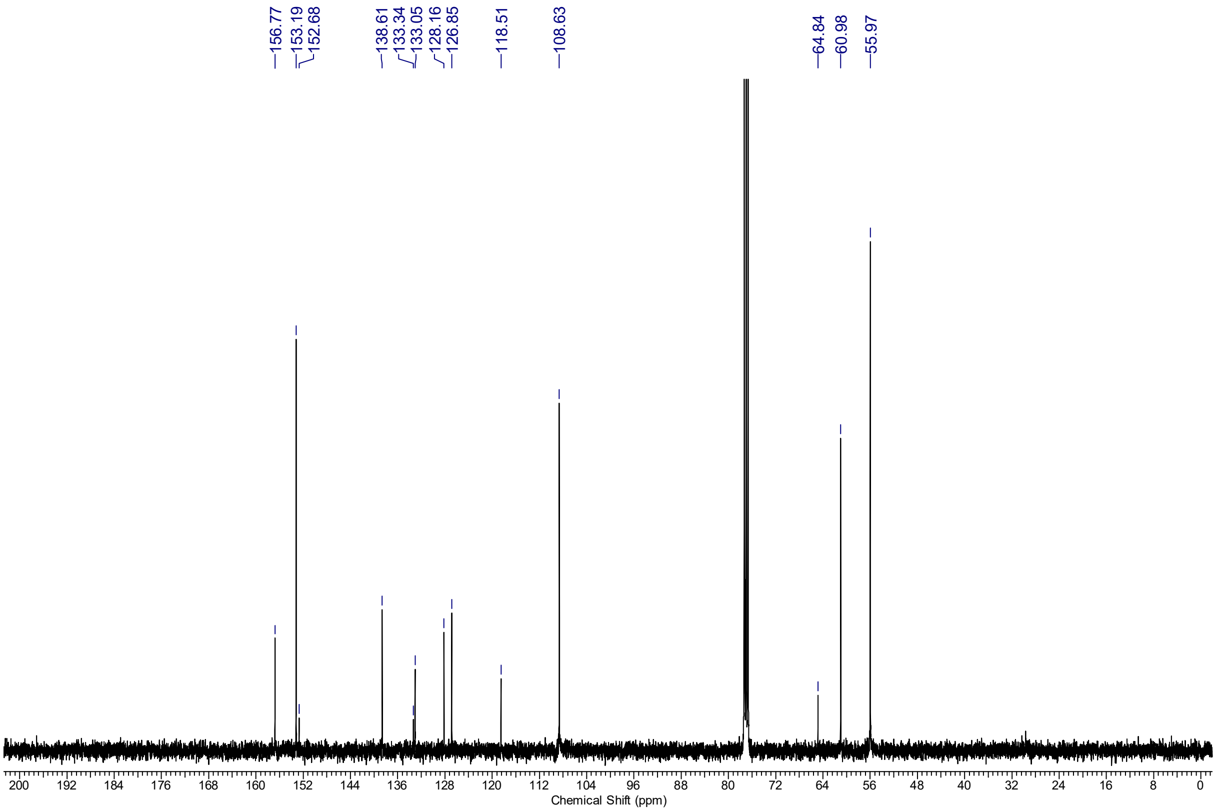

Figure S11. ^{13}C NMR (100.6 MHz) spectrum of **SubPzOH** in $CDCl_3$ at 298 K.

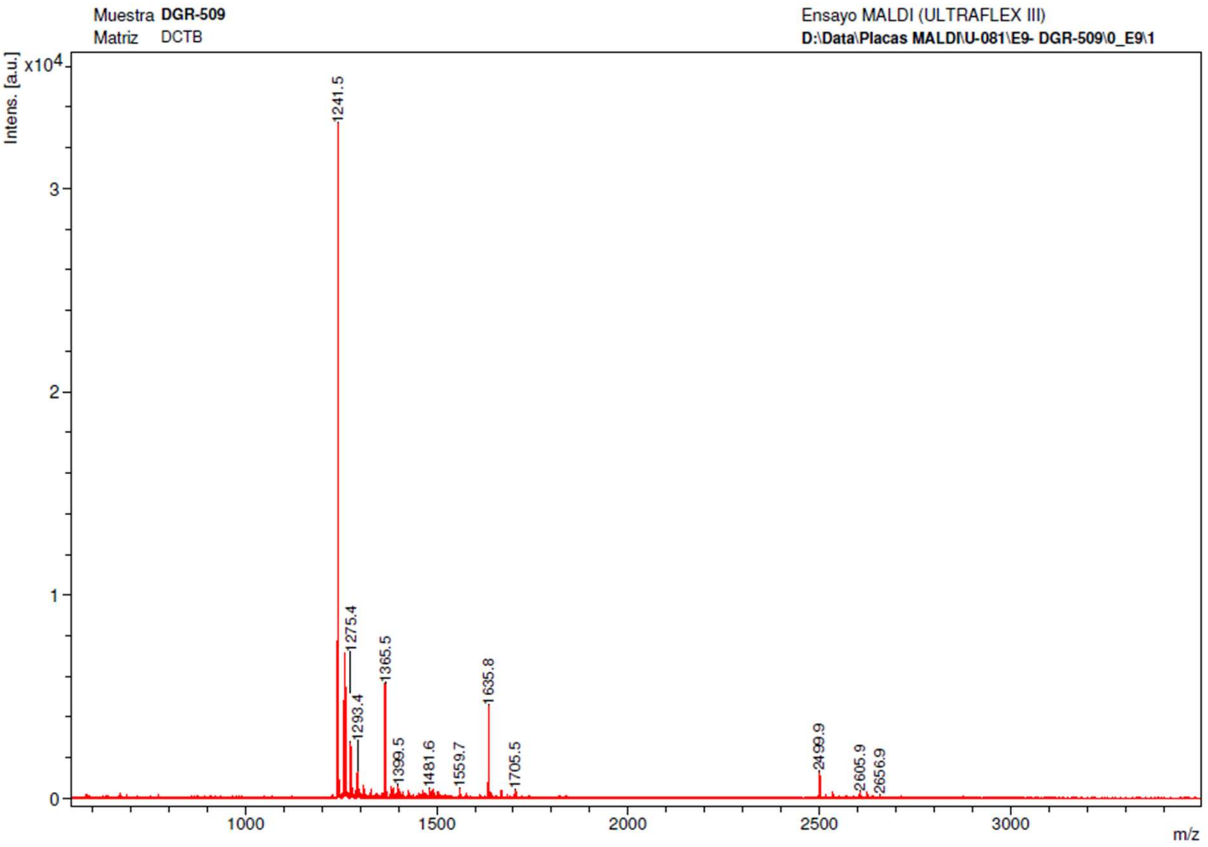

Figure S12. MALDI-TOF mass spectrum (in DCTB matrix) of **SubPzOH**.

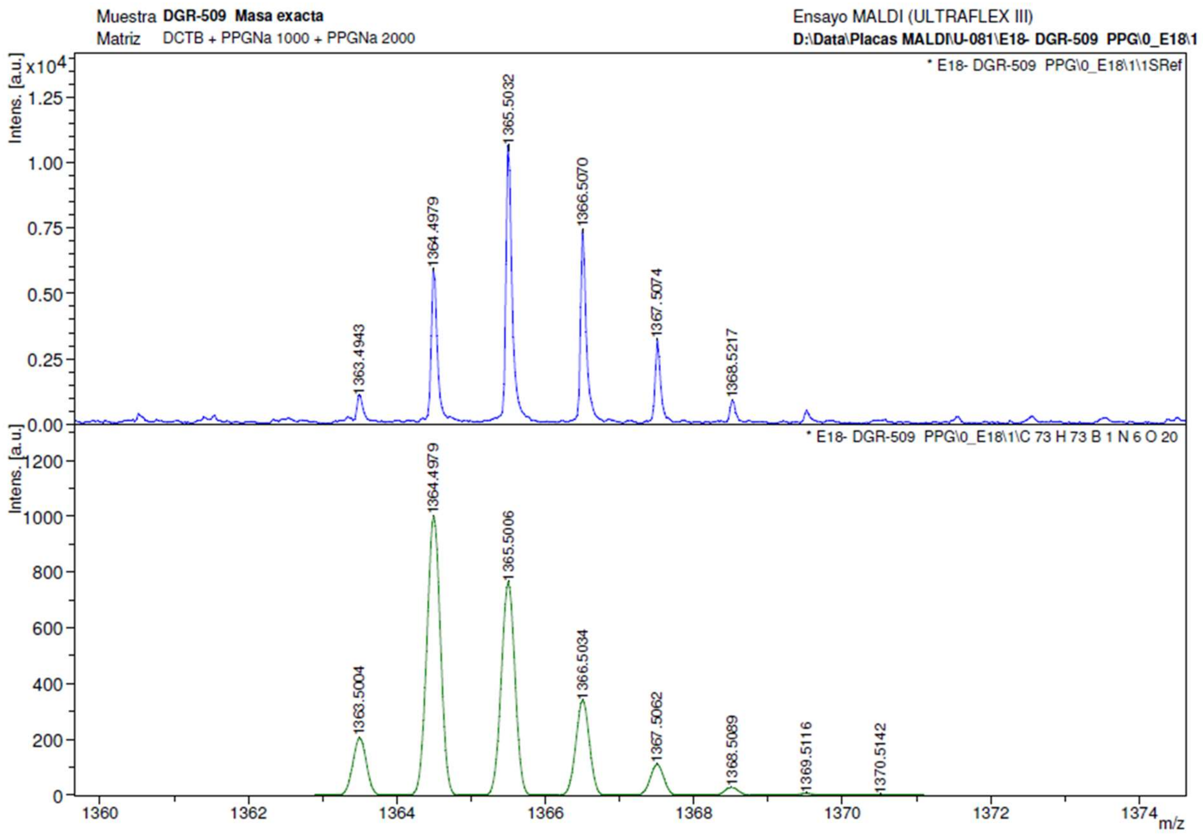

Figure S13. *Upper part* – HRMS (MALDI-TOF) spectrum (in DCTB + PPGNa 1000 + PPGNa 2000 matrix) of **SubPzOH**. *Lower part* – Calculated isotopic pattern of **SubPzOH**.

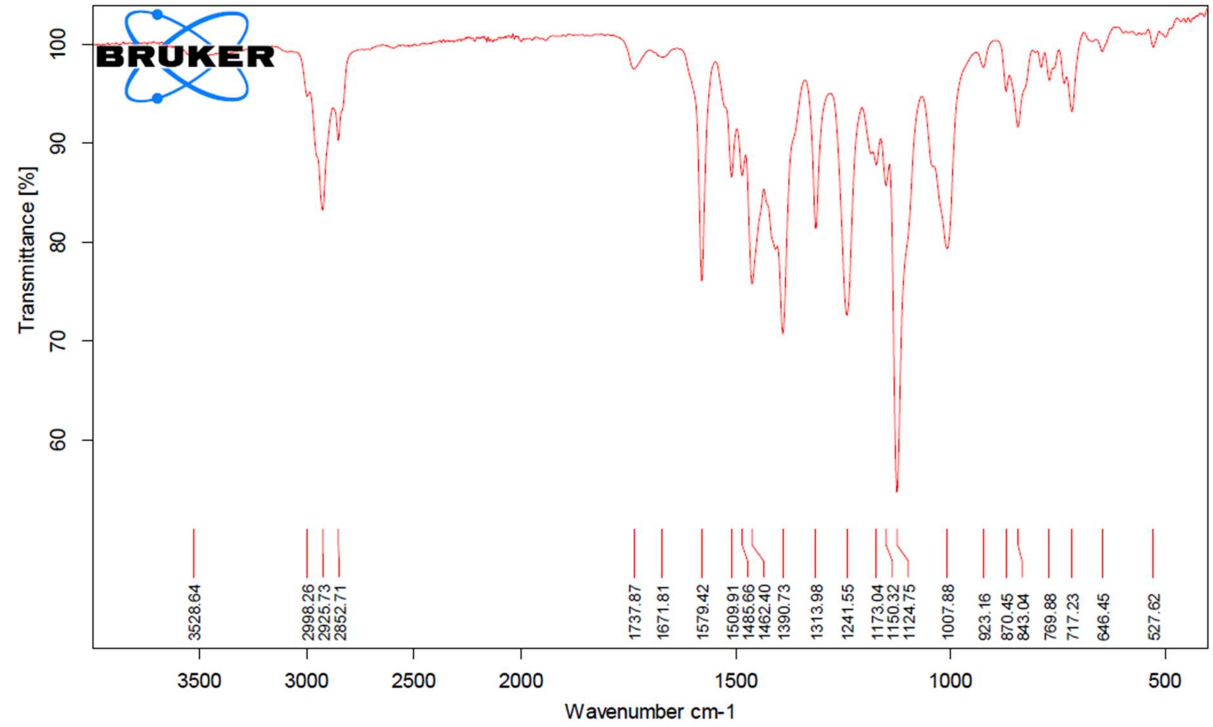

Figure S14. FT-IR spectrum (ATR) of **SubPzOH**.

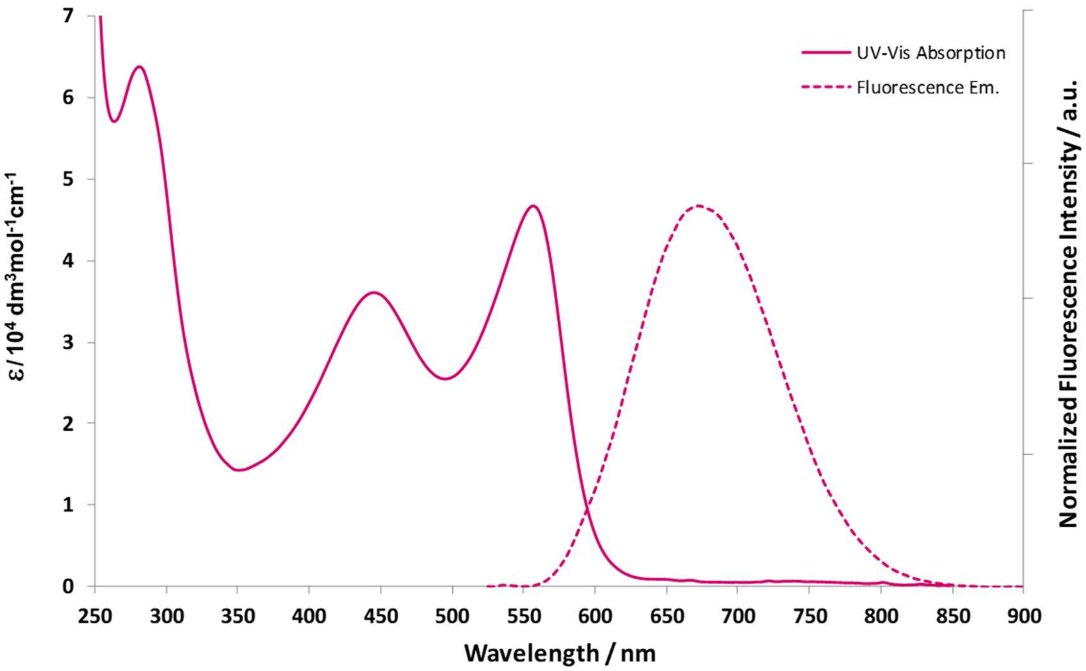

Figure S15. UV/Vis absorption (solid line) and normalized fluorescence (dashed line) spectra of **SubPzOH** in chloroform.

SubPzPnc₂

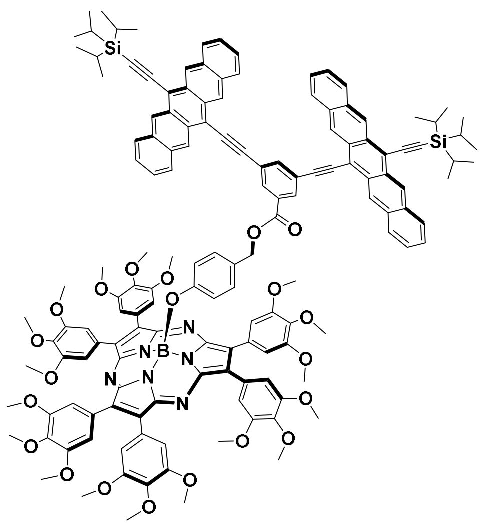

To a solution of **SubPzOH** (8.0 mg, 5.86 μmol), **Pnc₂COOH** (21.0 mg, 19.40 μmol), and DMAP (0.4 mg, 3.27 μmol) in deoxygenated THF (2 mL), EDC (4.0 mg, 26.00 μmol) was one-portion added under argon atmosphere at 0 ºC. The resulting reaction mixture was stirred at 0 ºC for 1 h, then the mixture was allowed to reach room temperature and kept under stirring overnight. Once the reaction is completed (monitored by TLC), the solvent was evaporated under reduced pressure, and the residue was passed through a short-pad of silica gel using a mixture of n-hexane/AcOEt/THF (2:6:2) as eluent. Then, the solvent was evaporated and the residue was purified by size-exclusion column chromatography on Bio-BeadsTM S-X1 (200-400 mesh) using chloroform as the eluent, to give the target material as a dark violet solid (2.8 mg, 20%).

^{1}H NMR (400 MHz, $CDCl_3$): δ(ppm) = 9.31 (s, 4H), 9.25 (s, 4H), 8.51 (s, 3H), 8.03 (d, *J* = 8.0 Hz, 4H), 7.97 (d, *J* = 8.0 Hz, 4H), 7.44–7.35 (m, 8H), 7.13 (d, *J* = 8.0 Hz, 2H), 7.12 (s, 12H), 5.58 (d, *J* = 8.0 Hz, 2H), 5.29 (s, 2H), 3.88 (s, 18H), 3.57 (s, 36H), 1.43–1.39 (m, 42H).

^{13}C NMR (100.6 MHz, $CDCl_3$): δ(ppm) = 156.9, 153.2, 138.6, 133.1, 132.3, 130.5, 130.3, 129.8, 128.7, 128.6, 126.8, 126.3, 126.2, 108.6, 61.0, 55.9, 19.0, 11.7.

FT-IR (ATR): ν(cm^{-1}) = 2930, 2860, 2128, 1726, 1580, 1510, 1461, 1390, 1313, 1240, 1177, 1126 (S), 1011, 875, 842, 768, 737, 674, 588, 460.

UV/Vis ($CHCl_3$) λ_{max} (nm) (log ε/dm^3 mol^{-1} cm^{-1}): 351 (4.7), 372 (4.7), 440 (4.6), 558 (4.7), 602 (4.5), 660 (4.7).

Fluorescence (CHCl3): λ_{ex} = 540 nm; λ_{em} = 674 nm.

MS (MALDI-TOF, DCTB, +): *m/z* = 2430.0 $[M]^+$

HRMS (MALDI-TOF): Calculated for $[M]^+$ $C_{150}H_{141}BN_6O_{21}Si_2$ 2427.9812; found 2427.9797.

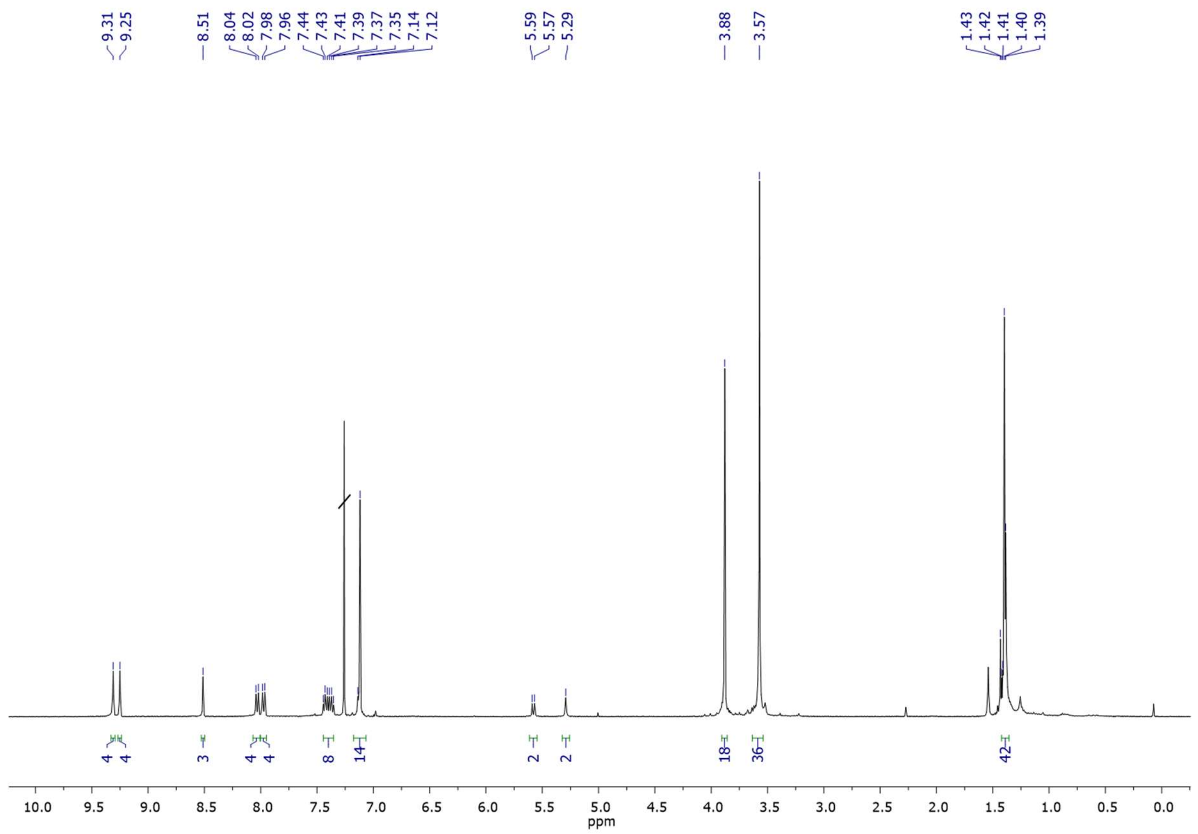

Figure S16. ^{1}H NMR (400 MHz) spectrum of **SubPzPnc$_2$** conjugate in $CDCl_3$ at 298 K.

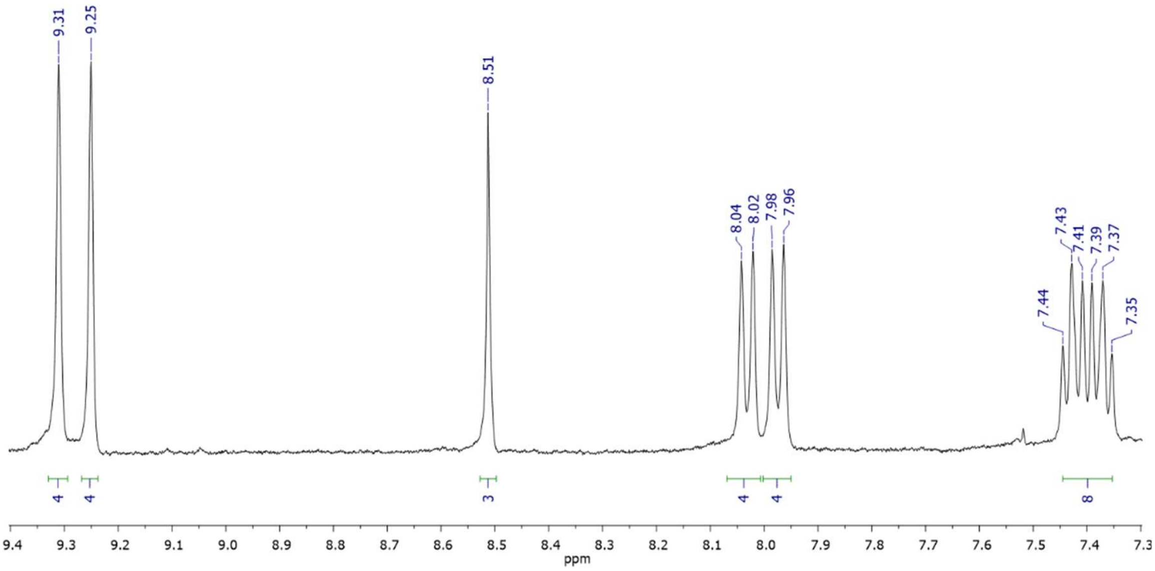

Figure S17. ^{1}H NMR spectrum of **SubPzPnc$_2$**, Expansion of the region between 9.4 ppm and 7.3 ppm (^{1}H aromatic pentacene dimer).

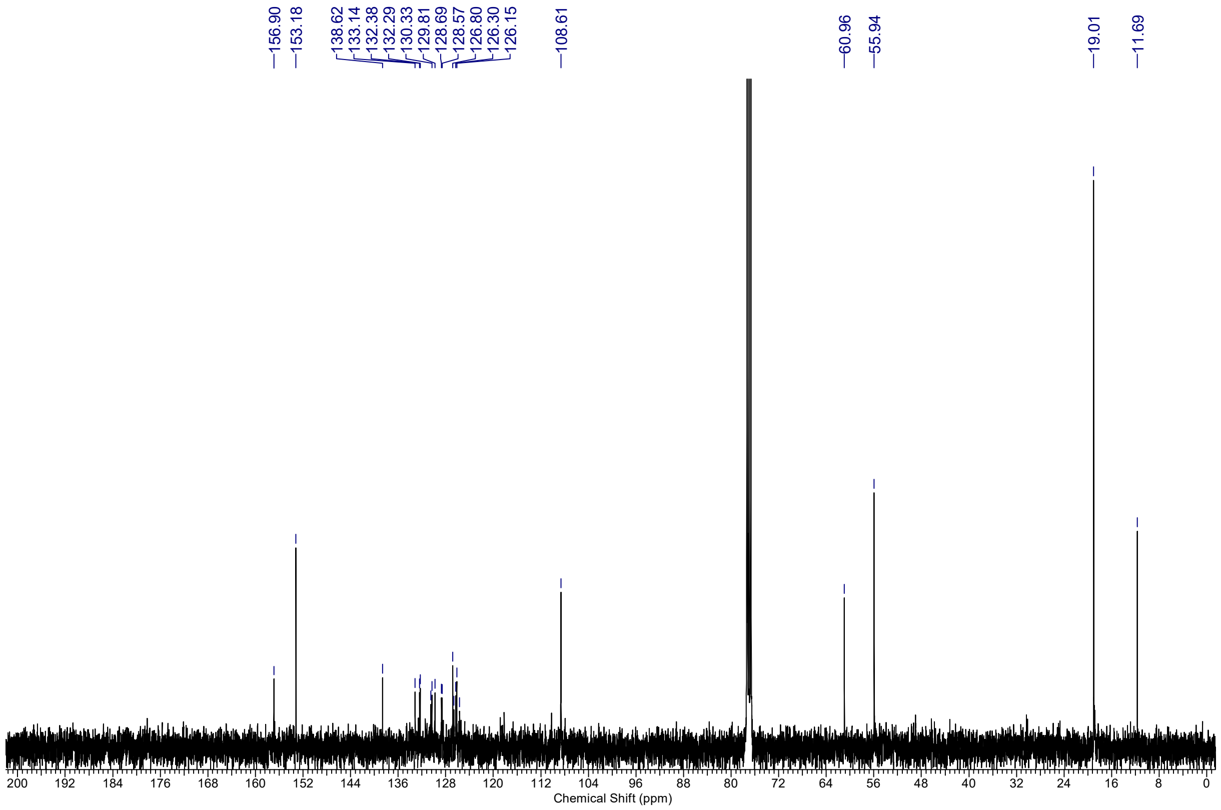

Figure S18. ^{13}C NMR (100.6 MHz) spectrum of **SubPzPnc₂** in $CDCl_3$ at 298 K.

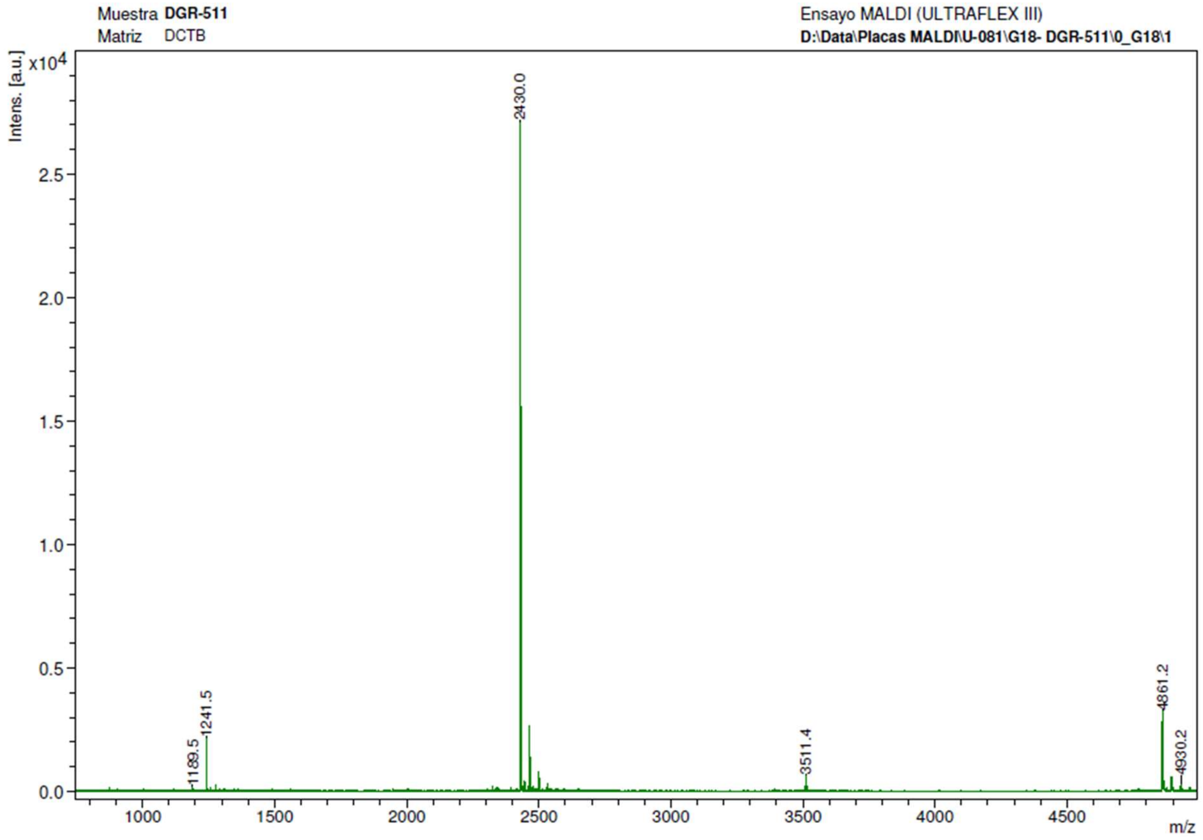

Figure S19. MALDI-TOF mass spectrum (in DCTB matrix) of **SubPzPnc$_2$**.

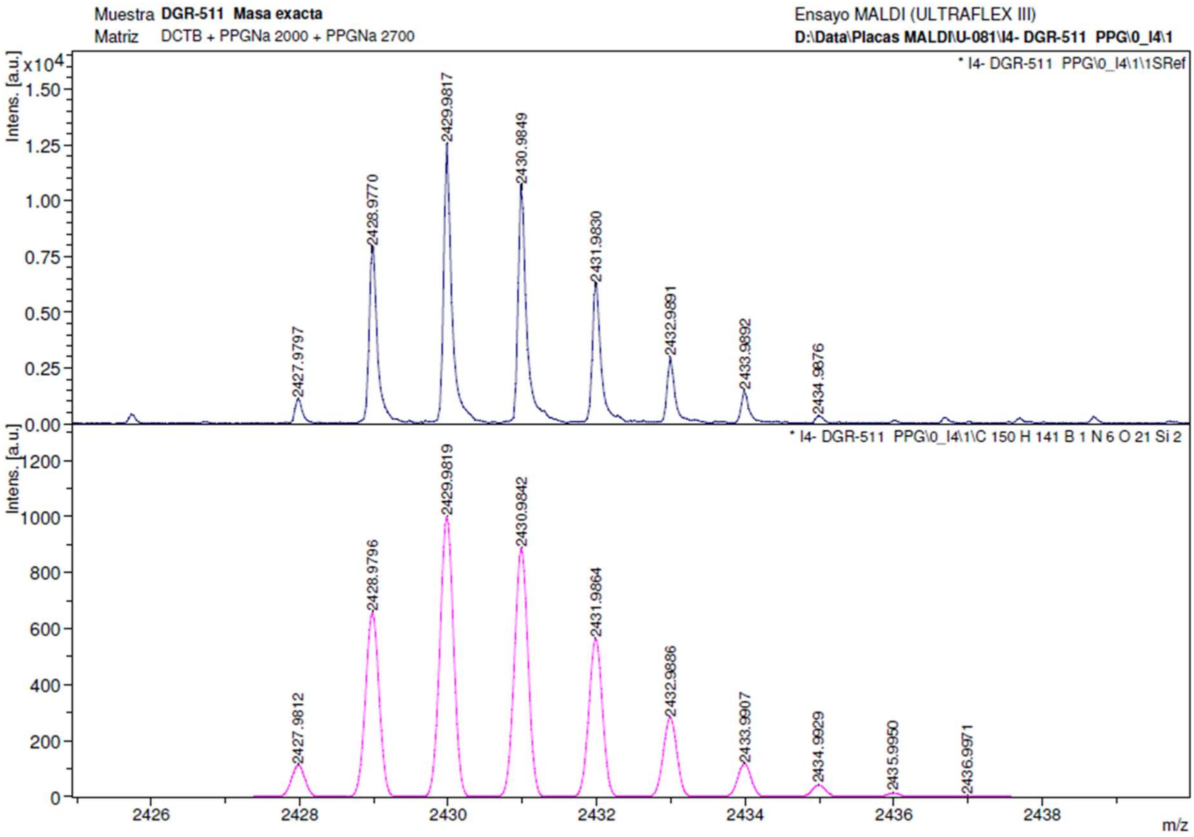

Figure S20. *Upper part* – HRMS (MALDI-TOF) spectrum (in DCTB + PPGNa 2000 + PPGNa 2700 matrix) of **SubPzPnc$_2$**. *Lower part* – Calculated isotopic pattern of **SubPzPnc$_2$**.

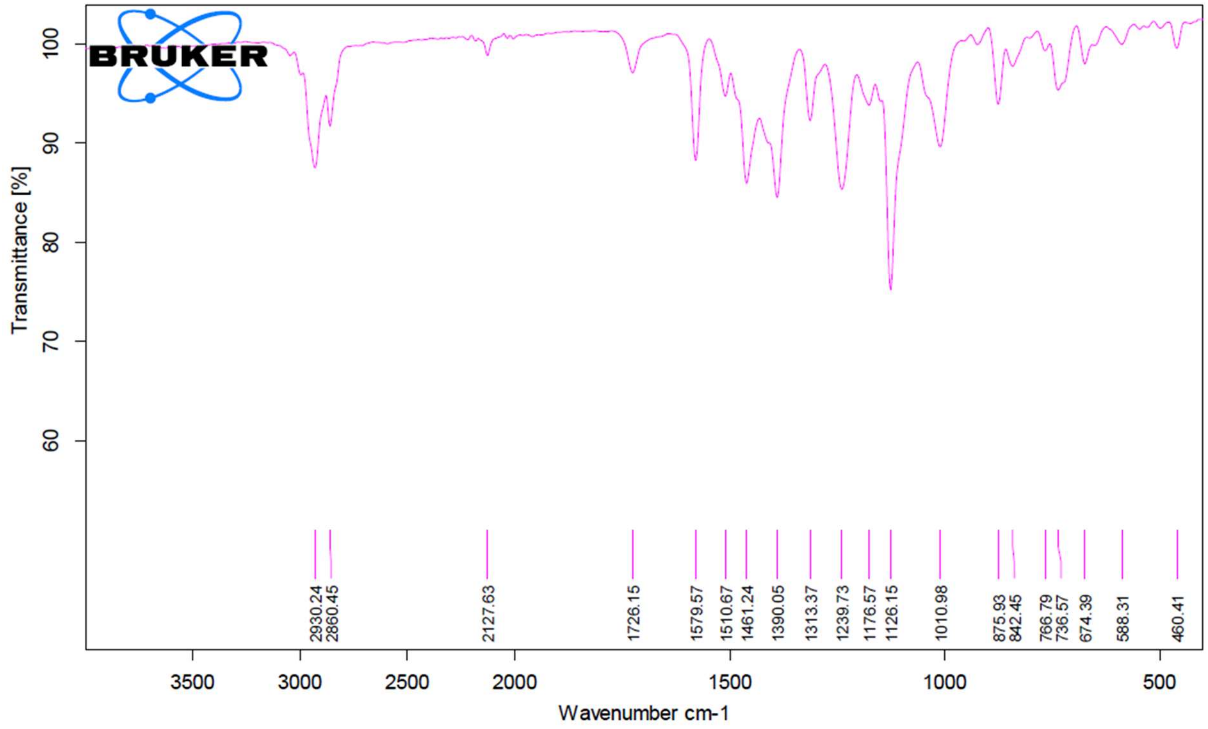

Figure S21. FT-IR spectrum (ATR) of **SubPzPnc$_2$**.

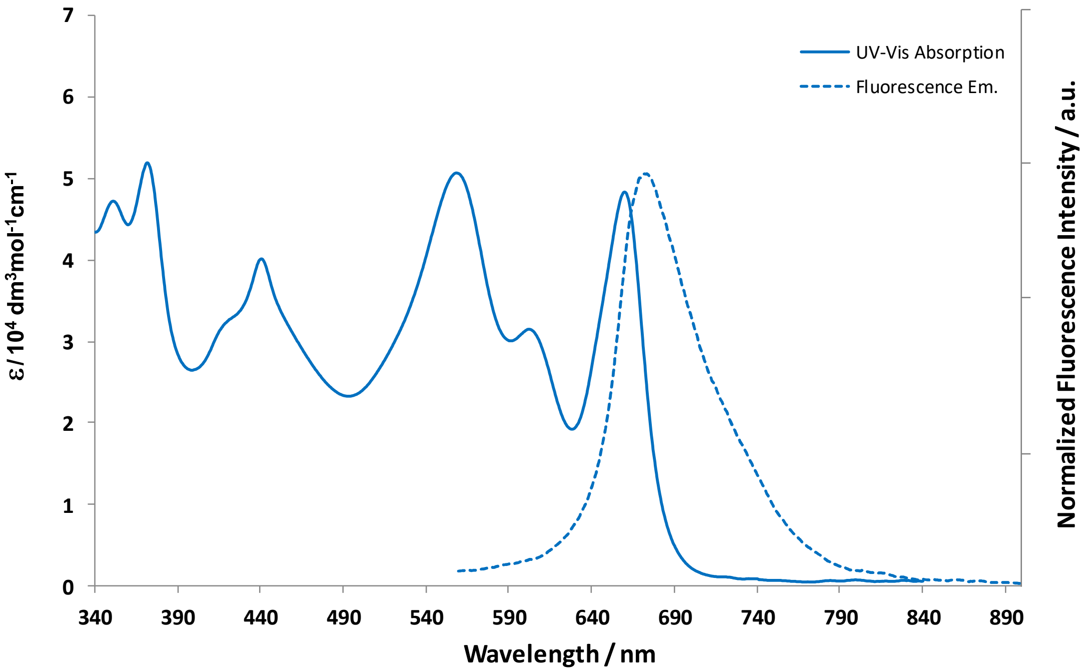

Figure S22. UV/Vis absorption (solid line) and normalized fluorescence (dashed line) spectra of **$SubPzPnc_2$** in chloroform.

Photophysics

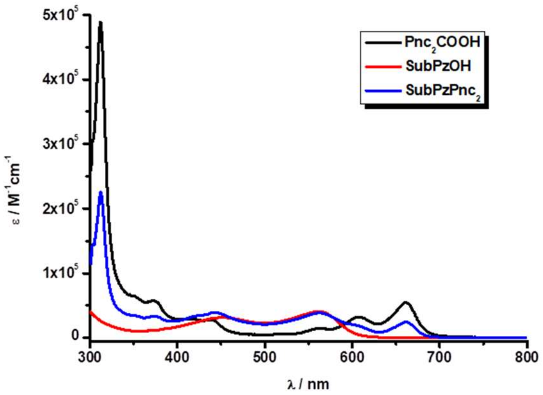

Figure S23. Absorption spectra of **Pnc_2COOH** (black), **SubPzOH** (red), and **$SubPzPnc_2$** (blue) in xylene (Xyl).

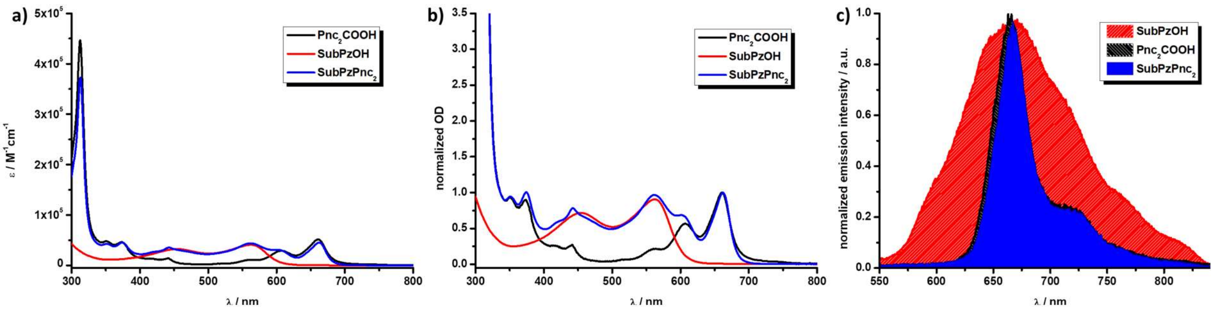

Figure S24. a) Absorption spectra of **Pnc_2COOH** (black), **SubPzOH** (red), and **$SubPzPnc_2$** (blue) in toluene. b) Normalized absorption spectra of **Pnc_2COOH** (black), **SubPzOH** (red), and **$SubPzPnc_2$** (blue) in toluene. c) Normalized fluorescence spectra of **SubPzOH** (red), **Pnc_2COOH** (black), and **$SubPzPnc_2$** (blue) in toluene. Normalization in (a): The **Pnc_2COOH** centered absorption (~660 nm) was used as a point of normalization for **Pnc_2COOH** and **$SubPzPnc_2$**, whereat the intensity of the **SubPzOH** centered maximum (~560 nm) was compared to **$SubPzPnc_2$**. Normalization in (b): The **Pnc_2COOH** centered fluorescence (~666 nm) was used as a point of normalization, thus highlighting the comparatively lower Φ_F of **SubPzOH** and **$SubPzPnc_2$**.

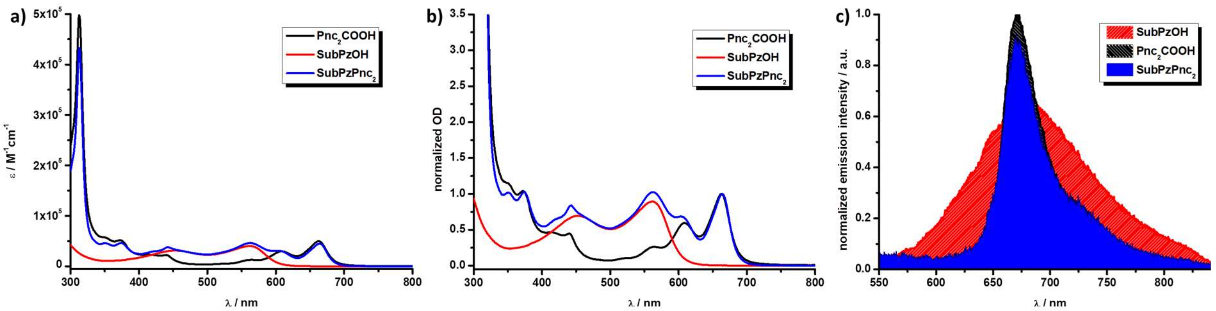

Figure S25. a) Absorption spectra of **Pnc₂COOH** (black), **SubPzOH** (red), and **SubPzPnc₂** (blue) in anisole. b) Normalized absorption spectra of **Pnc₂COOH** (black), **SubPzOH** (red), and **SubPzPnc₂** (blue) in anisole. c) Normalized fluorescence spectra of **SubPzOH** (red), **Pnc₂COOH** (black), and **SubPzPnc₂** (blue) in anisole. Normalization in (a): The **Pnc₂COOH** centered absorption (~660 nm) was used as a point of normalization for **Pnc₂COOH** and **SubPzPnc₂**, whereat the intensity of the **SubPzOH** centered maximum (~560 nm) was compared to **SubPzPnc₂**. Normalization in (b): The **Pnc₂COOH** centered fluorescence (~670 nm) was used as a point of normalization, thus highlighting the comparatively lower Φ_F of **SubPzOH** and **SubPzPnc₂**.

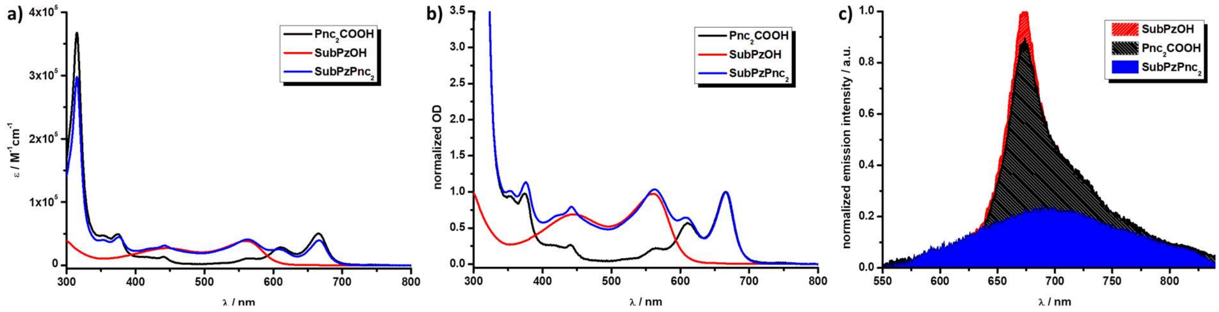

Figure S26. a) Absorption spectra of **Pnc₂COOH** (black), **SubPzOH** (red), and **SubPzPnc₂** (blue) in benzonitrile. b) Normalized absorption spectra of **Pnc₂COOH** (black), **SubPzOH** (red), and **SubPzPnc₂** (blue) in benzonitrile. c) Normalized fluorescence spectra of **SubPzOH** (red), **Pnc₂COOH** (black), and **SubPzPnc₂** (blue) in benzonitrile. Normalization in (a): The **Pnc₂COOH** centered absorption (~666 nm) was used as a point of normalization for **Pnc₂COOH** and **SubPzPnc₂**, whereat the intensity of the **SubPzOH** centered maximum (~560 nm) was compared to **SubPzPnc₂**. Normalization in (b): The **Pnc₂COOH** centered fluorescence (~674 nm) was used as a point of normalization, thus highlighting the comparatively lower Φ_F of **SubPzOH** and **SubPzPnc₂**.

Table S1. Photophysical parameters of **Pnc_2COOH**, **SubPzOH**, and **$SubPzPnc_2$** in toluene, anisole and benzonitrile.

Solvent	Compound	λ_{abs} / nm		λ_{em} / nm		Φ_F	τ_F / ps
		SubPz	Pnc_2	SubPz	Pnc_2	SubPz[a]	SubPz[a]
Toluene	Pnc_2COOH	–	660	–	666	0.016[b]	<200[b,c]
	SubPzOH	562	–	671	–	0.018	785
	$SubPzPnc_2$	562.5	662	–[d]	667	0.019[d]	<200[c,d]
Anisole	Pnc_2COOH	–	663	–	671.5	0.016[b]	<200[b,c]
	SubPzOH	561.5	–	679	–	0.0051	683
	$SubPzPnc_2$	563	664	–[d]	670.5	0.0059[d]	<200[c,d]
Benzonitrile	Pnc_2COOH	–	665.5	–	673	0.016[b]	<200[b,c]
	SubPzOH	560.5	–	688.5	–	0.0020	598
	$SubPzPnc_2$	562.5	666.5	–[d]	674	0.003[d]	<200[c,d]

[a]Values refer to the SubPz centered fluorescence if not stated otherwise. [b]Values refer to the Pnc_2 centered fluorescence. [c]Lifetime is below the resolution limit of our time-correlated single photon counting (TCSPC) setup. [d]SubPz centered fluorescence is not detectable / under the Pnc_2 centered emission in the conjugate.

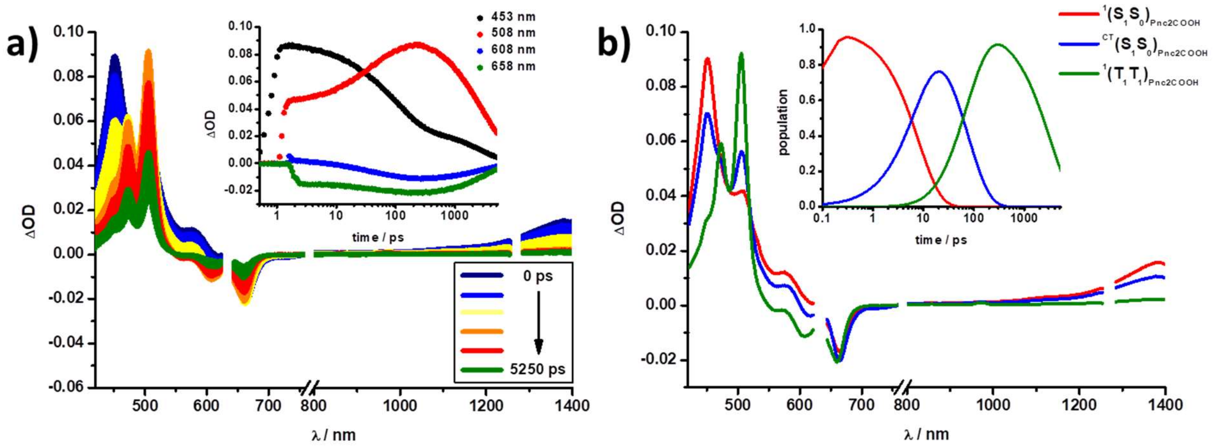

Figure S27. (a) Differential absorption spectra obtained from fsTA measurements (λ_{ex} = 633 nm) of **Pnc$_2$COOH** in xylene with several time delays between 0 and 5250 ps (colored solid lines) at rt; inset displays kinetics at 453, 508, 608, and 658 nm (colored dots). (b) Species associated spectra (SAS) of the fsTA data of **Pnc$_2$COOH** shown in a), with the initially formed **Pnc$_2$COOH** singlet excited state $^1(S_1S_0)_{Pnc2COOH}$ (red), intermediate state $^{CT}(S_1S_0)_{Pnc2COOH}$ (blue), and correlated triplet pair state $^1(T_1T_1)_{Pnc2COOH}$ (green); inset displays the relative populations of the respective SASs.

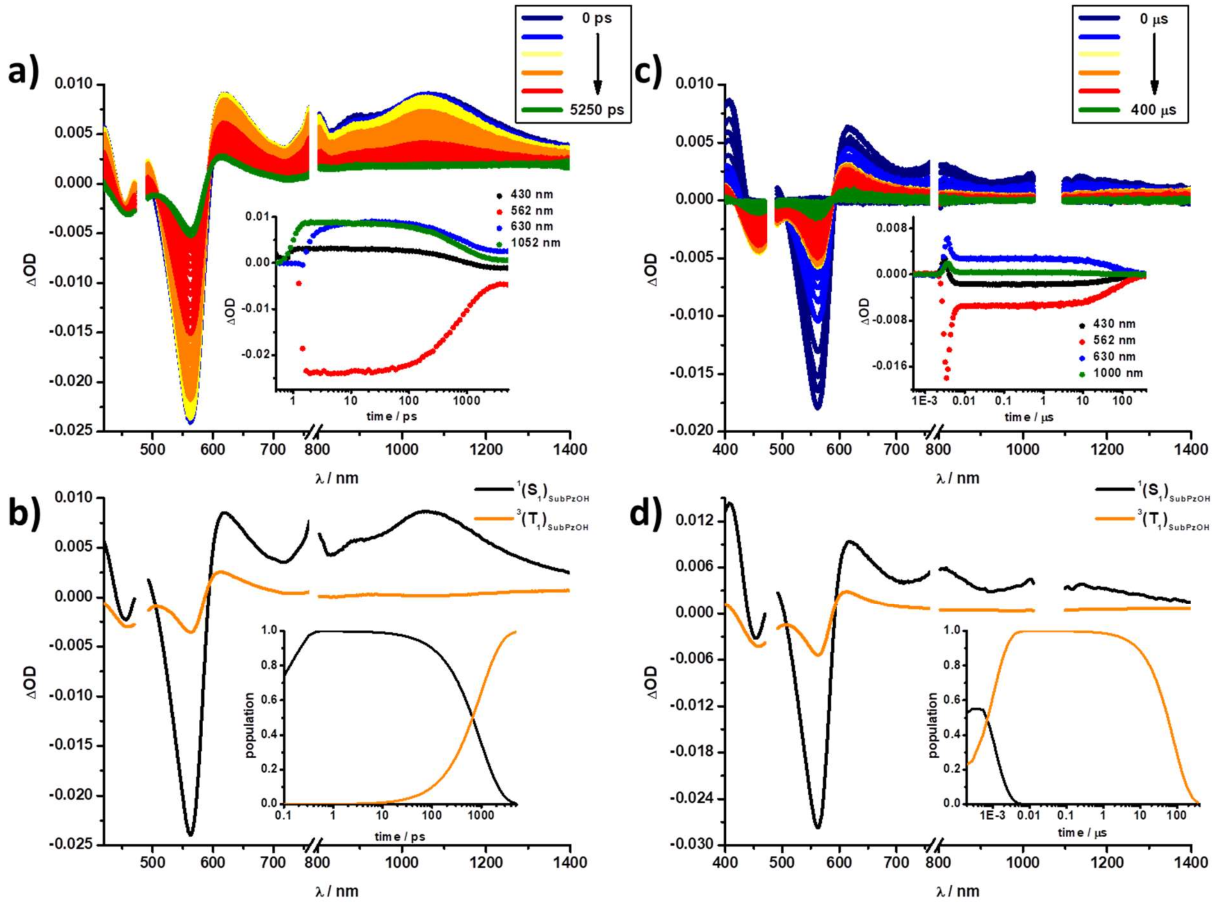

Figure S28. (a) Differential absorption spectra obtained from fsTA measurements (λ_{ex} = 480 nm) of **SubPzOH** in toluene with several time delays between 0 and 5250 ps (colored solid lines) at rt; inset displays kinetics at 430, 562, 630, and 1052 nm (colored dots). (b) Species associated spectra (SAS) of the fsTA data of **SubPzOH** shown in (a), with the initially formed singlet excited state $^1(S_1)_{SubPzOH}$ (black) and triplet excited state $^3(T_1)_{SubPzOH}$ (orange); inset displays the relative populations of the respective SASs. (c) Differential absorption spectra obtained from nsTA measurements (λ_{ex} = 480 nm) of **SubPzOH** in toluene with several time delays between 0 and 400 µs (colored solid lines) at rt; inset displays kinetics at 430, 562, 630, and 1000 nm (colored dots). (d) Species associated spectra (SAS) of the nsTA data of **SubPzOH** shown in (c), with the initially formed singlet excited state $^1(S_1)_{SubPzOH}$ (black) and triplet excited state $^3(T_1)_{SubPzOH}$ (orange); inset displays the relative populations of the respective SASs.

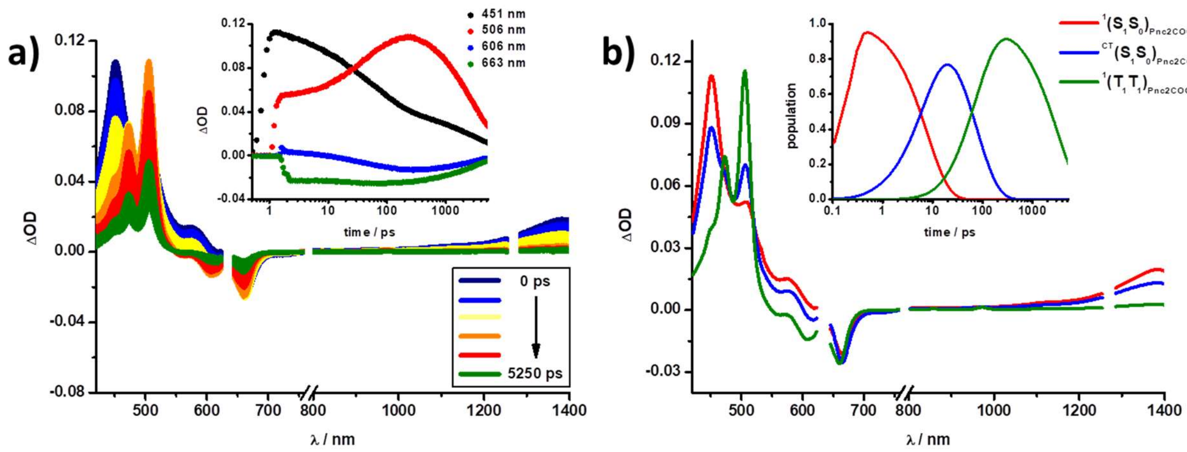

Figure S29. (a) Differential absorption spectra obtained from fsTA measurements (λ_{ex} = 633 nm) of **Pnc₂COOH** in toluene with several time delays between 0 and 5250 ps (colored solid lines) at rt; inset displays kinetics at 451, 506, 606, and 663 nm (colored dots). (b) Species associated spectra (SAS) of the fsTA data of **Pnc_2COOH** shown in a), with the initially formed **Pnc_2COOH** singlet excited state $^{1}(S_1S_0)_{Pnc2COOH}$ (red), intermediate state $^{CT}(S_1S_0)_{Pnc2COOH}$ (blue), and correlated triplet pair state $^{1}(T_1T_1)_{Pnc2COOH}$ (green); inset displays the relative populations of the respective SASs.

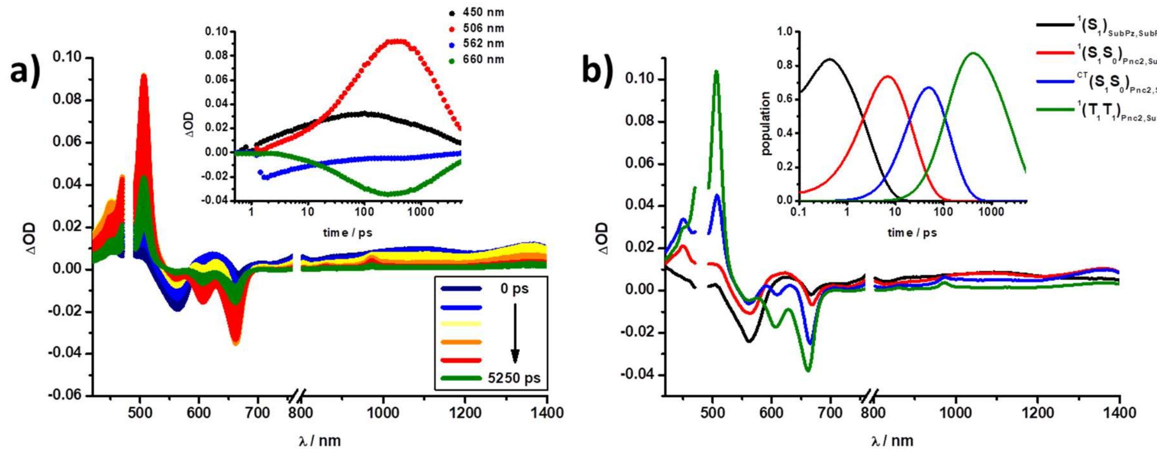

Figure S30. (a) Differential absorption spectra obtained from fsTA measurements (λ_{ex} = 480 nm) of **SubPzPnc₂** in toluene with several time delays between 0 and 5250 ps (colored solid lines) at rt; inset displays kinetics at 450, 506, 562, and 660 nm (colored dots). (b) Species associated spectra (SAS) of the fsTA data of **SubPzPnc₂** shown in (a), with the initially formed SubPz singlet excited state $^1(S_1)_{SubPz,SubPzPnc2}$ (black), the Pnc_2 singlet excited state $^1(S_1S_0)_{Pnc2,SubPzPnc2}$ (red), intermediate state $^{CT}(S_1S_0)_{Pnc2,SubPzPnc2}$ (blue), and correlated triplet pair state $^1(T_1T_1)_{Pnc2,SubPzPnc2}$ (green); inset displays the relative populations of the respective SASs.

Table S2. Kinetic data from fsTA and nsTA measurements and triplet quantum yields (TQYs) for **SubPzOH**, **Pnc_2COOH**, and **SubPzPnc$_2$** in toluene.

Compound	fsTA					nsTA	
	$^1(S_1)_{SubPz}$	$^1(S_1S_0)_{Pnc2}$	$^{CT}(S_1S_0)_{Pnc2}$	$^1(T_1T_1)_{Pnc2}$	TQY[a,b]	$^1(S_1)_{SubPz}$	$^3(T_1)_{SubPz}$
**SubPzOH**	955 ps	-	-	-	-	975 ps	80.06 µs
**Pnc_2COOH**	-	8.86 ps	78.57 ps	3.25 ns	135%	-	-
**SubPzPnc$_2$**	3.37 ps	26.55 ps	115.01 ps	2.88 ns	152%	n.r.[c]	-

[a]Determined *via* following the intensification of the transient bleaching of the Pnc_2 moiety and comparing the relative intensification of the species-associated for the $^{CT}(S_1S_0)_{Pnc2}$ and the $^1(T_1T_1)_{Pnc2}$ state, respectively. [b]An error margin in the range of ±10% may be considered when determining the TQYs. [c]n.r. = not resolvable. Lifetimes are either too long to be resolved with fsTA or too short to be resolved with nsTA.

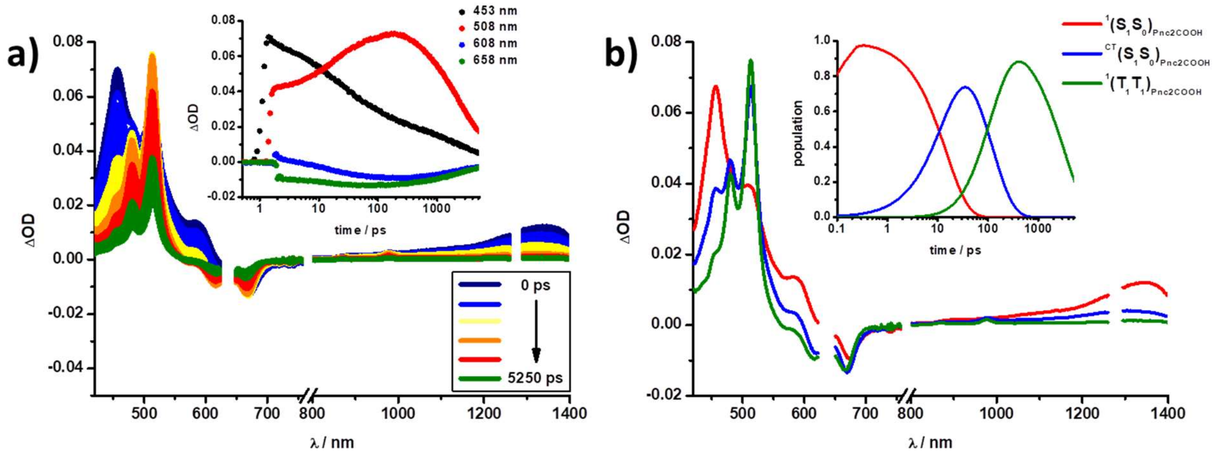

Figure S31. (a) Differential absorption spectra obtained from fsTA measurements (λ_{ex} = 633 nm) of **Pnc₂COOH** in anisole with several time delays between 0 and 5250 ps (colored solid lines) at rt; inset displays kinetics at 453, 508, 608, and 658 nm (colored dots). (b) Species associated spectra (SAS) of the fsTA data of **Pnc₂COOH** shown in a), with the initially formed **Pnc₂COOH** singlet excited state $^1(S_1S_0)_{Pnc2COOH}$ (red), intermediate state $^{CT}(S_1S_0)_{Pnc2COOH}$ (blue), and correlated triplet pair state $^1(T_1T_1)_{Pnc2COOH}$ (green); inset displays the relative populations of the respective SASs.

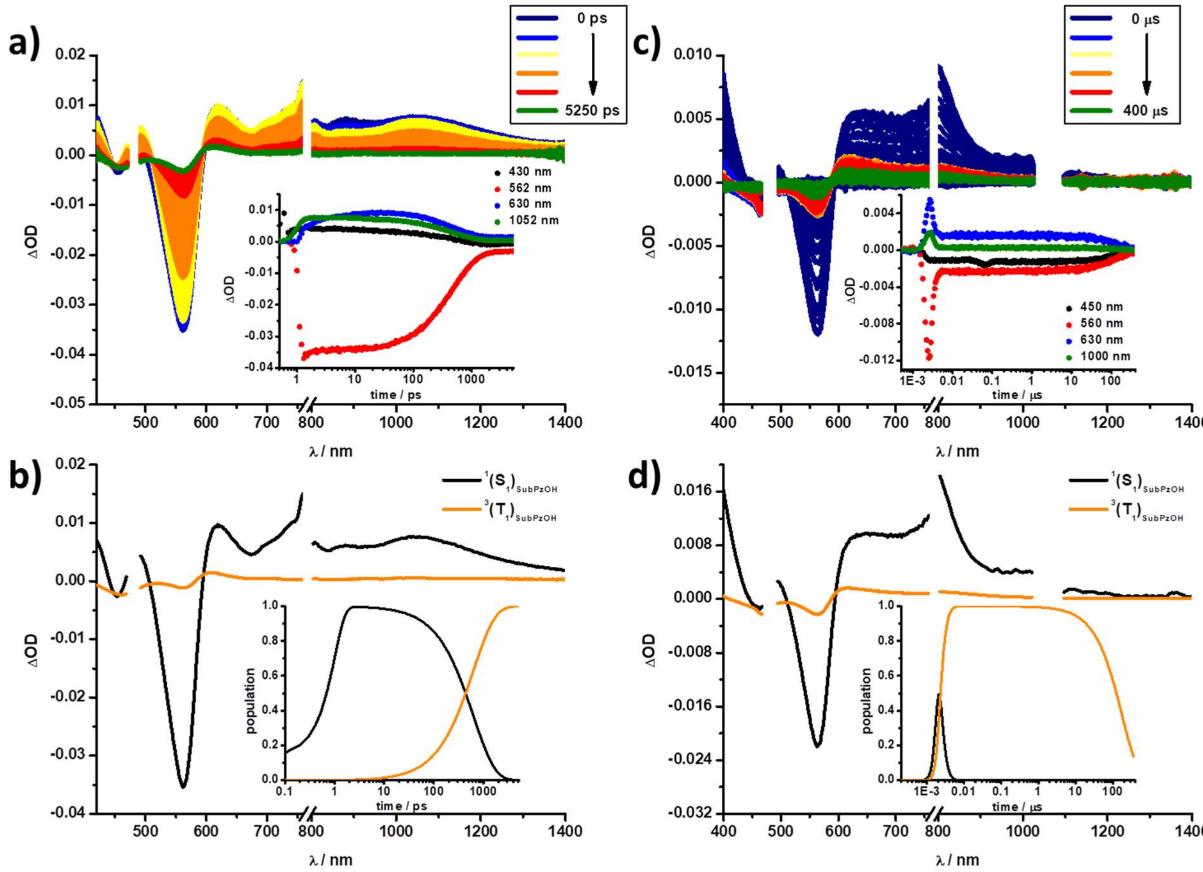

Figure S32. (a) Differential absorption spectra obtained from fsTA measurements (λ_{ex} = 480 nm) of **SubPzOH** in anisole with several time delays between 0 and 5250 ps (colored solid lines) at rt; inset displays kinetics at 430, 562, 630, and 1052 nm (colored dots). (b) Species associated spectra (SAS) of the fsTA data of **SubPzOH** shown in (a), with the initially formed singlet excited state $^1(S_1)_{SubPzOH}$ (black) and triplet excited state $^3(T_1)_{SubPzOH}$ (orange); inset displays the relative populations of the respective SASs. (c) Differential absorption spectra obtained from nsTA measurements (λ_{ex} = 480 nm) of **SubPzOH** in anisole with several time delays between 0 and 400 µs (colored solid lines) at rt; inset displays kinetics at 450, 560, 630, and 1000 nm (colored dots). (d) Species associated spectra (SAS) of the nsTA data of **SubPzOH** shown in (c), with the initially formed singlet excited state $^1(S_1)_{SubPzOH}$ (black) and triplet excited state $^3(T_1)_{SubPzOH}$ (orange); inset displays the relative populations of the respective SASs.

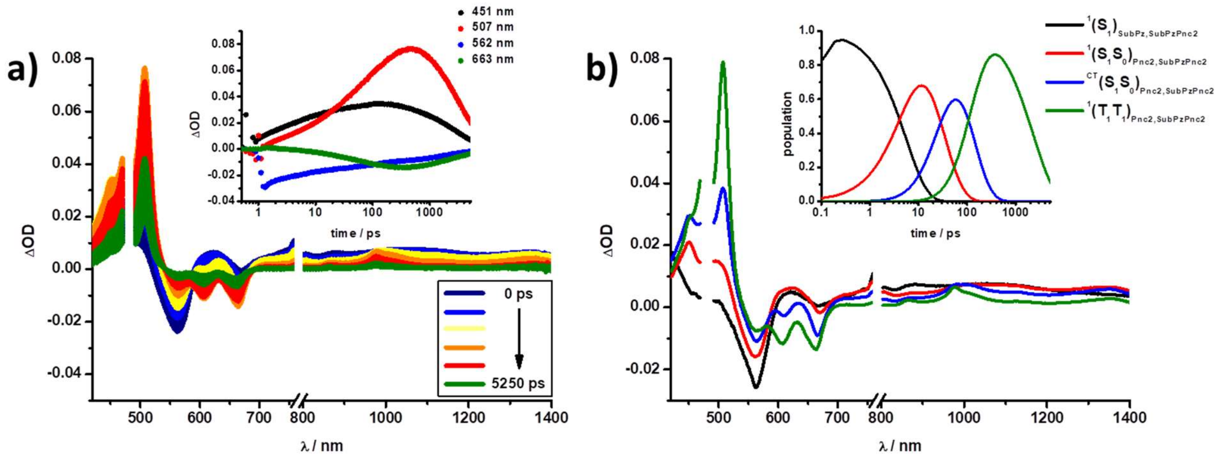

Figure S33. (a) Differential absorption spectra obtained from fsTA measurements (λ_{ex} = 480 nm) of **SubPzPnc$_2$** in anisole with several time delays between 0 and 5250 ps (colored solid lines) at rt; inset displays kinetics at 451, 507, 562, and 663 nm (colored dots). (b) Species associated spectra (SAS) of the fsTA data of **SubPzPnc$_2$** shown in (a), with the initially formed SubPz singlet excited state $^1(S_1)_{SubPz,SubPzPnc2}$ (black), the Pnc$_2$ singlet excited state $^1(S_1S_0)_{Pnc2,SubPzPnc2}$ (red), intermediate state $^{CT}(S_1S_0)_{Pnc2,SubPzPnc2}$ (blue), and correlated triplet pair state $^1(T_1T_1)_{Pnc2,SubPzPnc2}$ (green); inset displays the relative populations of the respective SASs.

Table S3. Kinetic data from fsTA and nsTA measurements and triplet quantum yields (TQYs) for **SubPzOH**, **Pnc_2COOH**, and **SubPzPnc_2** in anisole.

Compound	fsTA					nsTA	
	$^1(S_1)_{SubPz}$	$^1(S_1S_0)_{Pnc2}$	$^{CT}(S_1S_0)_{Pnc2}$	$^1(T_1T_1)_{Pnc2}$	TQY[a,b]	$^1(S_1)_{SubPz}$	$^3(T_1)_{SubPz}$
**SubPzOH**	630 ps	-	-	-	-	719 ps	180.00 μs
**Pnc_2COOH**	-	10.89 ps	105.54 ps	3.03 ns	137%	-	-
**SubPzPnc_2**	5.57 ps	25.94 ps	102.21 ps	2.30 ns	151%	n.r.[c]	-

[a]Determined *via* following the intensification of the transient bleaching of the Pnc_2 moiety and comparing the relative intensification of the species-associated for the $^{CT}(S_1S_0)_{Pnc2}$ and the $^1(T_1T_1)_{Pnc2}$ state, respectively. [b]An error margin in the range of ±10% may be considered when determining the TQYs. [c]n.r. = not resolvable. Lifetimes are either too long to be resolved with fsTA or too short to be resolved with nsTA.

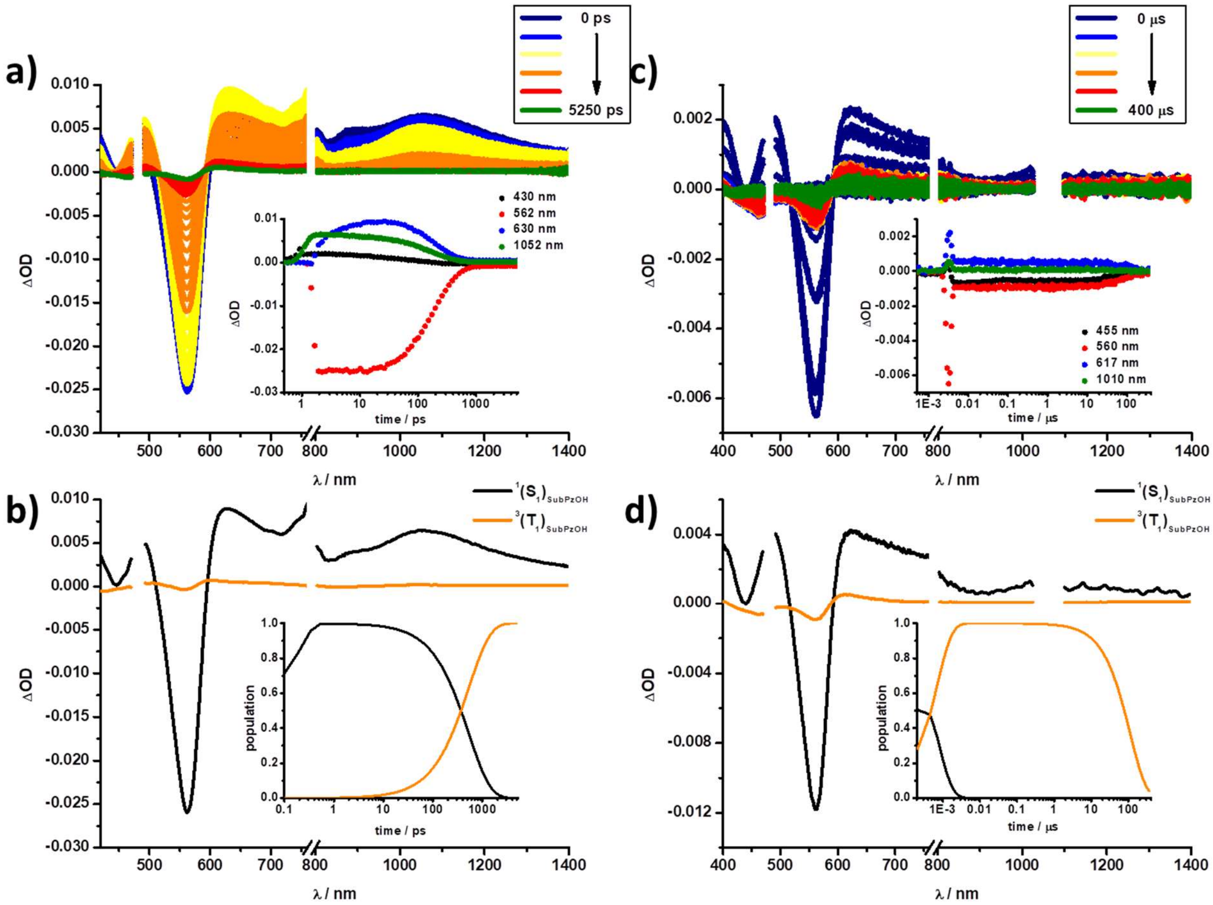

Figure S34. (a) Differential absorption spectra obtained from fsTA measurements (λ_{ex} = 480 nm) of **SubPzOH** in benzonitrile with several time delays between 0 and 5250 ps (colored solid lines) at rt; inset displays kinetics at 430, 562, 630, and 1052 nm (colored dots). (b) Species associated spectra (SAS) of the fsTA data of **SubPzOH** shown in (a), with the initially formed singlet excited state $^1(S_1)_{SubPzOH}$ (black) and triplet excited state $^3(T_1)_{SubPzOH}$ (orange); inset displays the relative populations of the respective SASs. (c) Differential absorption spectra obtained from nsTA measurements (λ_{ex} = 480 nm) of **SubPzOH** in benzonitrile with several time delays between 0 and 400 µs (colored solid lines) at rt; inset displays kinetics at 455, 560, 617, and 1010 nm (colored dots). (d) Species associated spectra (SAS) of the nsTA data of **SubPzOH** shown in (c), with the initially formed singlet excited state $^1(S_1)_{SubPzOH}$ (black) and triplet excited state $^3(T_1)_{SubPzOH}$ (orange); inset displays the relative populations of the respective SASs.

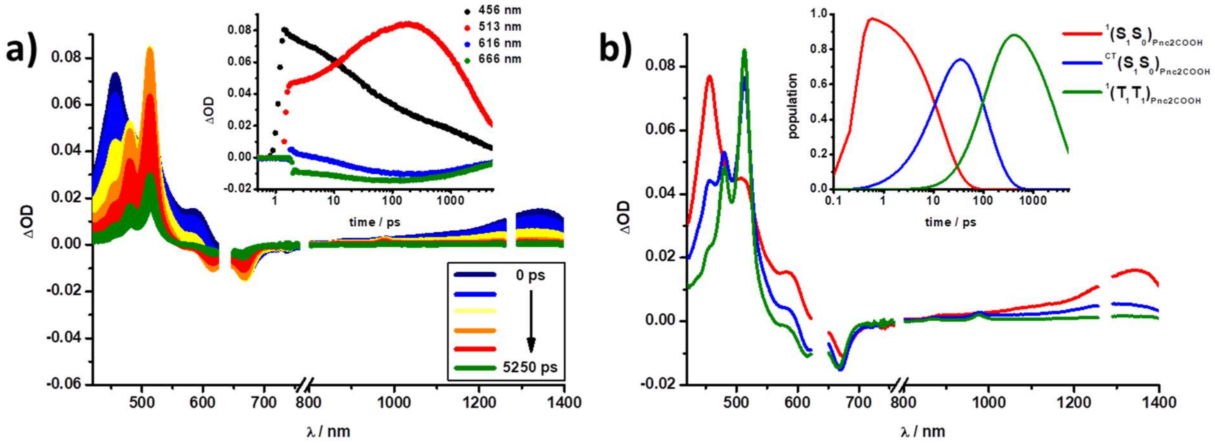

Figure S35. (a) Differential absorption spectra obtained from fsTA measurements (λ_{ex} = 633 nm) of **Pnc₂COOH** in benzonitrile with several time delays between 0 and 5250 ps (colored solid lines) at rt; inset displays kinetics at 456, 513, 616, and 666 nm (colored dots). (b) Species associated spectra (SAS) of the fsTA data of **Pnc_2COOH** shown in a), with the initially formed **Pnc_2COOH** singlet excited state $^1(S_1S_0)_{Pnc2COOH}$ (red), intermediate state $^{CT}(S_1S_0)_{Pnc2COOH}$ (blue), and correlated triplet pair state $^1(T_1T_1)_{Pnc2COOH}$ (green); inset displays the relative populations of the respective SASs.

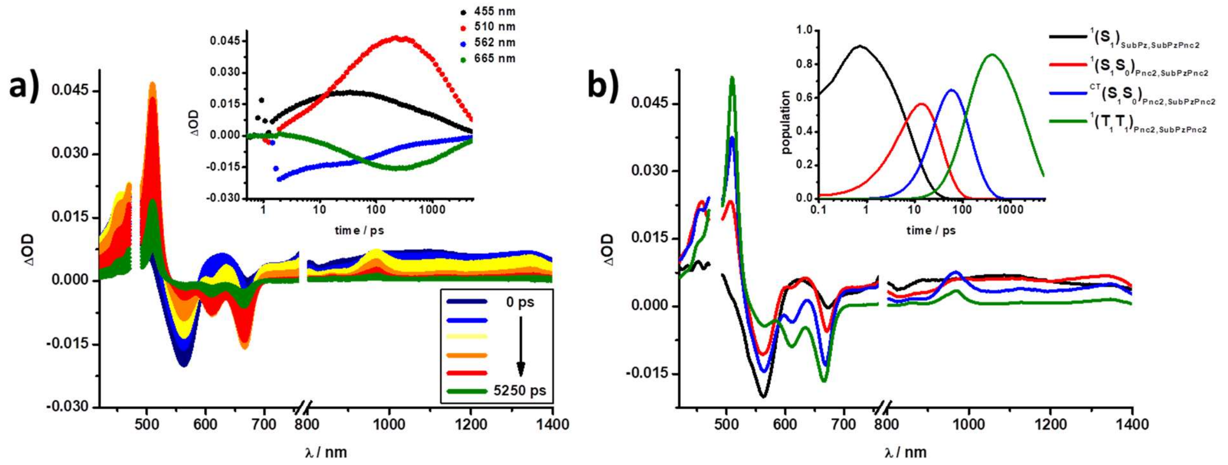

Figure S36. (a) Differential absorption spectra obtained from fsTA measurements (λ_{ex} = 480 nm) of **SubPzPnc$_2$** in benzonitrile with several time delays between 0 and 5250 ps (colored solid lines) at rt; inset displays kinetics at 455, 510, 562, 665 nm (colored dots). (b) Species associated spectra (SAS) of the fsTA data of **SubPzPnc$_2$** shown in (a), with the initially formed SubPz singlet excited state $^1(S_1)_{SubPz,SubPzPnc2}$ (black), the Pnc$_2$ singlet excited state $^1(S_1S_0)_{Pnc2,SubPzPnc2}$ (red), intermediate state $^{CT}(S_1S_0)_{Pnc2,SubPzPnc2}$ (blue), and correlated triplet pair state $^1(T_1T_1)_{Pnc2,SubPzPnc2}$ (green); inset displays the relative populations of the respective SASs.

Table S4. Kinetic data from fsTA and nsTA measurements and triplet quantum yields (TQYs) for **SubPzOH**, **Pnc_2COOH**, and **SubPzPnc$_2$** in benzonitrile.

Compound	fsTA					nsTA	
	$^1(S_1)_{SubPz}$	$^1(S_1S_0)_{Pnc2}$	$^{CT}(S_1S_0)_{Pnc2}$	$^1(T_1T_1)_{Pnc2}$	TQY[a,b]	$^1(S_1)_{SubPz}$	$^3(T_1)_{SubPz}$
**SubPzOH**	532.02 ps	-	-	-	-	632.00 ps	111.30 μs
**Pnc_2COOH**	-	14.52 ps	116.03 ps	3.24 ns	142%	-	-
**SubPzPnc$_2$**	8.65 ps	24.44 ps	116.09 ps	2.44 ns	161%	n.r.[c]	-

[a]Determined *via* following the intensification of the transient bleaching of the Pnc_2 moiety and comparing the relative intensification of the species-associated for the $^{CT}(S_1S_0)_{Pnc2}$ and the $^1(T_1T_1)_{Pnc2}$ state, respectively. [b]An error margin in the range of ±10% may be considered when determining the TQYs. [c]n.r. = not resolvable. Lifetimes are either too long to be resolved with fsTA or too short to be resolved with nsTA.

References

[1] A. Kunzmann, M. Gruber, R. Casillas, J. Zirzlmeier, M. Stanzel, M. W. Peukert, R. R. Tykwinski, D. M. Guldi, *Angew. Chem. Int. Ed.* **2018**, *57,* 10742–10747.

[2] P. Doppelt, S. Huille, *New J. Chem.* **1990**, *14*, 607–609.

[3] T. Higashino, M. S. Rodríguez-Morgade, A. Osuka, T. Torres, *Chem. Eur. J.* **2013**, *19*, 10353−10359.

[4] G. Lavarda, J. Zirzlmeier, M. Gruber, P. R. Rami, R. R. Tykwinski, T. Torres, D. M. Guldi, *Angew. Chem. Int. Ed.* **2018**, *57*, 16291–16295.

[5] D. P. Medina, I. Papadopoulos, G. Lavarda, H. Gotfredsen, P. R. Rami, R. R. Tykwinski, M. S. Rodríguez-Morgade, D. M. Guldi, T. Torres, *Nanoscale* **2019**, *11*, 22286–22292.

[6] M. A. Hink, N. V. Visser, J. W. Borst, A. van Hoek, A. J. W. G. V. Studies. *J. Fluoresc.* **2003**, *13*, 185–188.

[7] A value of $\kappa^2 = 2/3$ corresponds to a random orientation of the dipoles of the donor and acceptor (which is not expected to be fully appropriate to describe the real situation in the investigated conjugates). Nevertheless, since the value of κ^2 for conjugates is not known and the use of other values for κ^2 would just influence the magnitude of R_0 but not the overall picture, the calculations herein are based on a value of 2/3 for κ^2.

[8] L. Loura, *Int. J. Mol. Sci.* **2012**, *13*, 15252–15270.

[9] J. O'Neil Maryadele, *The Merck Index - An Encyclopedia of Chemicals, Drugs, and Biologicals*; Royal Society of Chemistry: Cambridge, 2013.

[10] C. Wohlfarth, *Refractive Indices of Pure Liquids and Binary Liquid Mixtures (Supplement to III/38)*; Springer-Verlag: Berlin Heidelberg, 2008.

[11] S. Budavari, *The Merck Index: An Encyclopedia of Chemicals, Drugs, and Biologicals*; Merck & Co Inc, 1989.
